# Quality and Functional Parameters of Fermented Milk Obtained from Goat Milk Fed with Broccoli and Artichoke Plant By-Products

**DOI:** 10.3390/foods11172601

**Published:** 2022-08-27

**Authors:** Raquel Muelas, Gema Romero, José Ramón Díaz, Paula Monllor, Juana Fernández-López, Manuel Viuda-Martos, Marina Cano-Lamadrid, Esther Sendra

**Affiliations:** Centro de Investigación e Innovación Agroalimentaria y Agroambiental (CIAGRO-UMH), Miguel Hernández University, Carretera de Beniel, 03312 Alicante, Spain

**Keywords:** thermophilic, yogurt, mesophilic, cheese, culture, revalorization, circular economy, health index, volatiles

## Abstract

Large amount of vegetal by-products are generated during production and processing steps. Introducing silage from vegetable by-products into dairy goat feed would be of great interest from the point of view of reducing costs and supporting the circular economy. The aim of this research was to study the effect of 40% inclusion of silage broccoli by-products and artichoke plant by-products in the diet of Murciano-Granadina goats throughout the lactation to establish milk suitability for fermented milks production. The novelty of this study is the use of milk from goats fed for a long term with a high inclusion of silages from artichoke plant and broccoli by-products, being the first one on broccoli inclusion. Two starter cultures thermophilic (YO-MIX^TM^300), and, mesophilic (MA400) were used and fermented milks were analyzed at two storage times after fermentation. Fermentation enhances antioxidant properties of fermented milks from all diets (*p* < 0.05), especially when mesophilic starter cultures are used. The main findings are that long term inclusion of 40% silage from broccoli and artichoke plant by-products in balanced diets of dairy goats yields milk suitable for fermentation by yogurt and cheese cultures, the inclusion of broccoli silage enhances antioxidant properties (*p* < 0.05), and, the inclusion of plant artichoke enhances fatty acids health indexes (*p* < 0.05).

## 1. Introduction

Broccoli (*Brassica oleracea italica*) and artichoke (*Cynara scolumus)* are highly relevant horticultural crops in Southern Spain (517,767 t and 199,944 t, production respectively in 2019), representing around 80% of the national production [1]. Both vegetables are either destined for the processing industries or for fresh consumption. The food industry is interested in the inflorescences of broccoli and in the flower bud of artichoke, being approximately 25% of its total biomass [2,3], so generating large volumes of by-products and, consequently, having a significant environmental impact. However, in Southern Spain, there is a long tradition of using by-products from broccoli and artichoke to feed sheep and goats, which, to some extent, contributes to reduce their environmental impact [3]. One drawback of using these fresh by-products as animal feed is their short shelf life due to their high moisture content. It is necessary to develop strategies to preserve theses sources of nutrients and allow their availability for longer periods. According to previous studies, these by-products have a good suitability for silaging that would allow their preservation over time without affecting their nutritional composition [4,5,6]. A key factor for their suitability as feed is to make sure that a long term inclusion of such silages in the diet (throughout a full lactation) does not have negative effects on the health and performance of the animal, nor on the composition of the obtained milk and technological characteristics of processed dairy products. From this point of view, research work has recently been carried out to establish the optimal inclusion of broccoli and artichoke plant by-product silage on isoproteic and isoenergetic diets, formulated for milking Murciano-Granadina goats. Some information on their effect on milk production, composition, functional properties and technological characteristics in milk has been already reported [5,6,7,8,9]. The use of silages from vegetable by-products in the diet of dairy goats would be of great interest for reducing feeding costs and supporting the circular economy. Goat milk production in Spain in 2019 was 475,630 t, representing 45% of the total produced by the European Mediterranean countries [1]. Ninety percent of the milk is transformed by the dairy industry; 64% into cheese and 20% for the production of dairy products such as fermented milks. The consumption of fermented milks in Spain in 2020 was around 600 million kg (15.03 L per capita in 2020) [10].

Goat milk is characterized by having a higher nutritional value and health properties than cow’s milk as a result of its greater digestibility, mineral bioavailability, protein and fatty acids profile which have been associated with immunological and antibacterial properties [11,12]. However, due to its characteristic goaty flavor, mainly provided by short-chain fatty acids: caproic acid (C6:0), caprylic acid (C8:0) and capric acid (C10:0), some consumers find goat milk unacceptable [11]. This goaty flavor can be mitigated with the fermentation process due to the development of flavors provided by a complex mixture of aromatic compounds [13] formed through the metabolic pathways of lactic acid bacteria (LAB). Lactose fermentation, fat lipolysis and proteolysis are responsible for the release of organic acids, free fatty acids (FFA) that can be metabolized to secondary alcohols and fatty acid lactones, and free amino acids and precursors of other aromatic compounds [14,15,16,17,18]. Such flavor complexity is able to mask to some extent the goaty flavor [19] and so to improve the acceptability of goat milk products.

Commonly, in-doors raised dairy goats are fed conventional feed based on concentrates, and the obtained milk is used for yogurt and fermented milks making. There is a clear tendency to enhance the functional properties of fermented dairy foods, one example is the enrichment of fermented milks by directly adding plant extracts to improve their functional properties [20,21,22,23]. 

Commercial lyophilized yogurt cultures are mixtures of thermophilic starter cultures, mainly *Streptococcus thermophilus* and *Lactobacillus delbrueckii subsp. bulgaricus*. These microorganisms cooperate with each other: exchanging metabolites, improving the growth rate, the size of each microbial population and the acidification rate [24,25,26,27]. Likewise, for the production of soft, semi-cured and cured cheeses, as well as butter or buttermilk mainly mesophilic cultures are used. Some common cultures are mixtures made of *Lactoccoccus *(*Lactococcus lactis subsp. lactis*, *Lactococcus lactis subsp. cremoris and Lactococcus lactis subsp. Lactis biovar diacetylactis*), mesophilic microorganisms, characterized by good acidifying activities, formation of exopolysaccharides, proteolytic capacity and aroma production due to the formation of diacetyl flavor compounds by the citrate metabolism of *Lactococcus lactis subsp. Lactis biovar diacetylactis*, and also may include thermophilic bacteria (*Streptococcus thermophilus*) to achieve even faster lactic acid production during cheese making [28].

The present work was run in parallel to that by other authors [7,8,29] which were focused on animal health and welfare, milk yield, quality and composition. The present study focuses on the obtained dairy products: fermented milks. The aim of the present research is to study the effect of 40% inclusion of broccoli and artichoke by-products in the diet of Murciano-Granadina goats throughout the lactation (preexperimental, early lactation, middle lactation and late lactation) to establish milk suitability for fermented milks production. Two starter cultures (YO-MIX^TM^ 300 and MA400) were used and fermented milks were analyzed at two storage times after fermentation. Viability of microorganisms, pH, syneresis, chemical composition, antioxidant activity, nutritional/health fatty acid indexes and volatile profile in fermented milks were determined. It is important to highlight that previous research about quality and technological properties of fermented milk from short term inclusion and low percentage of inclusion in the diet of artichoke by-products (up to 25%) was published by Muelas et al. (2017) [6]. The present is the first study using milk from goats fed for a long term (5 months) with a high (40%) inclusion of silages from artichoke plant and fresh broccoli by-products, being the first one on broccoli inclusion. 

## 2. Materials and Methods

### 2.1. Animals, Facilities and Dietary Treatments

The present study has been carried out in the teaching and research farm with Murciano-Granadina goats located at the Orihuela Higher Polytechnic School at the Miguel Hernández University of Elche. Goat selection criteria (average body weight: 44.6 ± 7.81 kg; average milk yield: 2.43 ± 0.21 kg/day; average somatic cell count: 5.14 ± 0.55 Log cells/mL), division of the animals and housing were those established as previously published [8]. A total of 72 multiparous goats were selected, classified into three homogeneous groups and housed in three independent in-door yards, with access to outdoor patios (size calculated to provide at least 2.30 m^2^/animal). The dimensions of the in-doors yards were calculated based on free access to the feeders (35 cm/goat) and at least 1.50 m^2^/goats for the free movement of the animals. This study was approved by the Ethical Committee of Experimentation of the Miguel Hernández University (code UMH.DTA.GRM.01.15). At the beginning of the experiment, all goat groups were fed a conventional diet (group 1: CD1; group 2: CD2 and group 3: CD3) based on 2.5 kg/animal/day of a single ration of alfalfa hay and a mixture of grains and pellets and straw ad libitum. This first period lasted 4 weeks and was considered as the pre-experimental period (PE). From that period on, the inclusion of differentiated diets began. The three groups characterized by the administered feeding were: group 1 with control diet (CD), which represents the conventional diet; group 2 (BD) with the inclusion of 40% broccoli by-product silage in the diet, and, group 3 (APD) with the inclusion of 40% artichoke plant by-product silage in the diet. In all cases, the diets were formulated in an iso-energetic and iso-proteic form with an average of 38.77, 163.33 and 370.33 g/Kg dm (Dry Matter) for ether extract, crude protein and neutral detergent fibre respectively. The daily intake, formulation and nutritional parameters analyzed for each of the diets are reflected in the research article previously published [8]. 

### 2.2. Milking Sampling

Each group (CD; BD; APD) of goats (*n* = 24) were milked separately once a day in a Casse-type milking parlor with a low-line milking machine (2 × 12 × 12, Gea-Farm Technologies^®^, Bönen, Germany) and the milk was stored independently in three refrigeration tanks (Gea Farm Technologies Iberica SL, Germany) at temperature between 4–6 °C. At each sampling time, 4 L of each tank were collected (bottled in 1 Liter Pyrex^®^ bottles) on two consecutive days: (3 tanks × 4 L (2 L for each tested culture) × 2 days = 24 L). Milk samplings took place at week 4th (PE: pre-experimental), 12th (EL: early lactation), 17th (ML: Middle Lactation) and 22nd (LL: Late Lactation). 

### 2.3. Starter Cultures

Two commercial mixed starter cultures (freeze-dried power) were used: the thermophilic yogurt starter culture YO-MIX^TM^ 300 (Danisco, Spain; *Streptococcus thermophilus* and *Lactobacillus delbrueckii subsp. bulgaricus*) and the mesophilic cheese/butter starter culture MA400 (Danisco, Spain; *Lactococcus lactis subsp. Lactis*, *Lactococcus lactis subsp. Cremoris*, *Lactococcus lactis subsp. Lactis biovar diacetylactis*, and, *Streptococcus thermophilus*). Lyophilized cultures were suspended in sterile peptone solution and after allowing some minutes for rehydration the suspended cultures were inoculated to tempered milk according to manufacturer’s indications dosage.

### 2.4. Fermented Milks (FMs) Manufacturing

Elaborations were performed at lactation weeks 4th (PE), week 12th (EL), week 17th (ML) and week 22th (LL). Milk (in 1 L Pyrex^®^ bottles) was pasteurized at 80 °C for 30 min and cooled in an ice-water bath until it reached a temperature of approximately 43 °C for the inoculation of thermophilic yogurt cultures and 30 °C for mesophilic cheese cultures. Milk was aseptically distributed in 100 mL sterile polypropylene bottles, inoculated with the starter culture and incubated under the optimal conditions for each of the cultures specified by the technical data sheet (43 °C for YO-MIX^TM^ and 30 °C for MA400). The pH was determined just before the inoculation of the starter culture and monitored until reaching an approximate pH of 4.8, and then, FMs were stored at 4 °C to subsequently carry out the corresponding analysis. Fermented milks were evaluated at 2 (T2) and 30 (T30) days of refrigerated storage to determine the number of viable microorganisms, pH, composition, syneresis, polyphenol content, and antioxidant activity. Samples of FMs were frozen at −20 °C to determine sugars, lactic acid, volatile compounds and fatty acids profile. The total number of fermented milk batches was 48 (4 lactation period × 2 repetitions × 3 goat group × 2 cultures = 48 batches). 

### 2.5. Raw Milk and Fermented Milk Analysis

#### 2.5.1. Determination of Culture Viability and pH

For microbiological counts of fermented milks serial dilutions were prepared in sterile peptone water and de Man Rososa Sharpe (MRS) and M17 agar (Merck KGaA, Darmstadt, Germany) were used for enumeration of *Lactobacillus* spp. and *Streptococcus* spp./*Lactococcus* spp. respectively. Milks fermented with YO-MIX^TM^300 were analyzed for lactobacilli in MRS (incubated under microaerophilic conditions at 37 °C for 48 h) and streptoccoci in M17 (aerobic conditions at 37 °C for 24 h). Milk cultured with MA400 was analyzed for streptocci/lactocci counts on M17 under the same conditions.

pH was determined at 25 °C using a pH Basic 20 instrument (Crison, Barcelona, Spain). Three replicates were run for pH and microbial counts.

#### 2.5.2. Milk, Fermented Milk and Whey Compositional Analysis

The chemical composition (fat, protein, lactose and total solids) of milk was determined by means of a near-infrared spectrophotometer (FOSS, Milko-Scan FT120; Foss Electric, Hiller∅d, Denmark) using the base calibration of the equipment (Foss Electric validated according to ISO 21543:2006 (ISO, 2006). The equipment software contains different modules with calibrations for precise determinations of milk components (Improved Milk Calibration, Foss Electric, 1999), fermented milks (Application Note N° 94, P/N 492264, Foss Electric, 1999) and whey (pH Independent Whey Calibration, Application Note No. 91, P/N 491928, Foss Electric, 1999). The determination in raw milk was made directly on the sample. Fermented milk samples, in order to reduce their viscosity were diluted 1/3 with zero-liquid (Cero S-6060, Hiller∅d, Denmark) before analysis. Whey samples were obtained by centrifuging the curd using the methodology previously described [6,30] that allowed syneresis determination and whey separation for its compositional analysis. Syneresis (% drained whey) was expressed as whey/milk ratio mass [14]. Analysis were performed in duplicate in samples warmed at 40 °C. 

#### 2.5.3. Sugar Profile and Lactic Acid

The individual analysis of sugars and lactic acid in raw milk took 5 mL of milk and the methodology previously described was used [31]. However, in fermented milks, the methodology described by Pablo Mortera et al., 2018 [32] was used with some modifications, 5 g of sample were weighed and 10 mL of ultrapure water were added. In both cases, the sample was homogenized for approximately 1 min and centrifuged at 11,000 rpm for 20 min at a temperature of 4 °C. In both cases, prior to chromatographic analysis, the supernatant was filtered through a 0.45 micron nylon filter (Merck KGaA, Darmstadt, Germany) and injected into a high performance liquid chromatograph Hewlett-Packard HP-1100, Woldbronn, Germany). Chromatographic analysis was performed in isocratic gradient with a flow of 0.5 mL/min and a mobile phase consisting of ultrapure water acidified with 0.1% phosphoric acid. The column used was a Supelcogel C-610H, 30 cm × 7.8 mm (Supelco Park, Bellefonte, PA, USA). Sugars were detected with a refractive index detector and lactic acid with a diode array (DAD) at a wavelength of 210 nm. The quantification was carried out using external calibration curves prepared with pure standards of sugars and lactic acid (Merck KGaA, Darmstadt, Germany). Analysis were performed in duplicate.

#### 2.5.4. Total Phenol Content (TPC), and Antioxidant Activity 

The determination of the TPC content was carried out using the Folin-Ciocalteu method previously described [33]. For quantification, 5 concentrations of gallic acid (50, 100, 150, 200 and 250 mM) were prepared and the results were expressed in mg allic acid equivalents (mg GAE/L). For the determination of the antioxidant capacity, the ABTS and DPPH assays were performed ([34,35], respectively). Antioxidant activity was expressed as mM Trolox/mL as calculated from a Trolox calibration curve (0.15, 0.30, 0.5, 0.75 and 1 mM). 

#### 2.5.5. Health Indexes from Fatty Acids Profile

Regarding the analysis of the fatty acid profile for its subsequent calculation of the healthy indexes, the extraction method described by Romeu-Nadal et al. (2004) with some modifications was followed [36]. Fatty acid methylation was performed according to Nudda et al. (2005) [37], also with some modifications. Fatty acid methyl esters (FAMEs) were separated using a chromatograph with FID detector (GC-17A Shimadzu, Kyoto, Japan) equipped with a capillary column (CP Sil 88 100 m × 0.25 mm, 0.20 µm particle size, Agilent, Santa Clara, CA, USA). The injector and detector temperatures were 240 °C and 250 °C, respectively. The programmed temperature was 45 °C for two minutes, increasing to 165 °C in a range of 8 °C/min, maintained at 165 °C for 35 min, increasing to 210 °C in a range of 5.5 °C/min. The fatty acids were individually identified by comparison with the relative retention times of a standard mix of external standards (37FAME mix, Supelco, Bellefonte, PA, USA). The nutritional/healthy indexes from fatty acids profile were calculated following the formulas compiled previously [16,38]. The nutritional/healthy indexes were: PUFA/SFA (Polyunsaturated Fatty Acids/Saturated fatty acids, Equation (1)), MUFA/SFA (Monounsaturated Fatty Acid/Saturated fatty acids, Equation (2)), n6/n3 (omega 6/omega (3), Equation (3)), LA/ALA (Linoleic Acid/α-Linolenic acid, Equation (4)), Oleic acid/Stearic acid (Equation (5), ∑CLA/Vaccenic acid (Equation (6)), IA (Aterogenicity index, Equation (7)), IT (thrombogenicity index, Equation (8)), HFA (hypercholesterolemic index, Equation (9)), HH (Hypocholesterolemic/Hypercholesterolemic ratio, Equation (10)), HPI (Health promoting index, Equation (11)), DI14 (Desaturation index of C14:0, Equation (12)), DI16 (Desaturation index of C16:0, Equation (13)), and DI18 (Desaturation index of C18:0, Equation (14)).

(1) ΣPUFA/ΣSFA

(2) ΣMUFA/ΣSFA

(3) $$\left(C18:2n-6+\;C18:3n-6+\;C20:2n-6+\;C20:3n-6+\;C20:4n-6\right)/\left(C18:3n-3\right)$$

(4) C18:2n6/C18:3n3

(5) C18:1/C18:0

(6) CLAc9t11+ CLAt9c11+ CLAt12,14/C18:1t11

(7) $$\left[C12:0+\left(4\times \;C14:0\right)+\;C16:0\right]/\Sigma \mathrm{UFA};\;\mathrm{UFA}:\;\mathrm{Undaturated}\;\mathrm{Fatty}\;\mathrm{acids}\;\left(\mathrm{MUFA}+\mathrm{PUFA}\right)\;$$

(8) $$\left(C14:0+\;C16:0+\;C18:0\right)/\left[\left(0.5\times \Sigma \mathrm{MUFA}\right)+\left(0.5\times \Sigma n-6\;\mathrm{PUFA}\right)+\left(3\times \Sigma n-3\;\mathrm{PUFA}\right)+\left(n-3/n-6\right)\right]$$

(9) ∑C12:0+ C14:0+ C16:0

(10) $$\left(\mathrm{cis}-C18:1+\;\Sigma \mathrm{PUFA}\right)/\left(C12:0+\;C14:0+\;C16:0\right)$$

(11) $$\Sigma \mathrm{UFA}/\left[C12:0+\left(4\times \;C14:0\right)+\;C16:0\right]$$

(12) C14:1cis/C14:0

(13) ∑C16:1t4; C16:1t5; C16:1t9; C16:1t10; C16:1t11; C16:1c7; C16:1c9; C16:1c10; C16:1c11/C16:0

(14) ∑C18:1t4; C18:1t5; C18:1t6,8; C18:1t9; C18:1t10; C18:1t11; C18:1t12; C18:1t13,14; C18:1t16; C18:1c9; C18:1c11; C18:1c12; C18:1c13; C18:1c16/C18:0

#### 2.5.6. Volatile Compounds

Fermented milks from the pre-experimental period (all groups in conventional diet) and from late lactation (different diets) were analyzed for volatile profile. To determine the volatile profile and its quantification, headspace solid phase microextraction (HSPME) of volatiles was performed [39] using a SPME 50/30 mm DVB/CAR/PDMS (Divinylbenzene/Carboxen/Polydimethylsiloxane) fiber (Supelco). The fiber was mounted in an automatic injection port AOC 6000 Plus Auto Sampler (Shimadzu Scientific Instruments, Inc., Columbia, MD, USA). Five grams of fermented milk and one gram of salt were introduced in each vial. The sample was tempered at 40 °C during 5 min. Afterwards the sample was exposed to the fiber for 50 min under stirring. After equilibration the fiber was desorbed for 3 min at the injection port of a chromatograph Shimadzu GC2030 coupled with a Shimadzu TQ8040 NX mass spectrometer detector (Shimadzu Scientific Instruments, Inc., Columbia, MD, USA) with a SLB-5 MS column (Teknokroma, Barcelona, Spain) (30 m length, 0.25 mm internal diameter, 0.25 μm film thickness). The parameters of the detector were: (i) mass range 35–400 m/z, (ii) scan speed 5000 amu/s, (iii) event time of 0.200 s, and (iv) electronic impact of 70 eV. Helium was used as a carrier gas, with a split ratio of 1:10, a purge flow of 6 mL min^−1^, and a total column flow of 0.8 mL min^−1^. The temperature of the detector was 300 °C, and the temperature of the injector was 220 °C. Chromatographic conditions were initial temperature 40 °C for 2 min, temperature gradient of 10 °C/min up to 200 °C and maintaining 200 °C for 10 min. minutes. Peak identification was performed by comparing the retention times of the standard compounds and the Wiley library spectra.

### 2.6. Statistical Analysis

A general linear model (Proc. GLM, SAS 9.4, 2012) was used to study separately each of the studied cultures (YO-MIX^TM^ 300 and MA400) and each of the four lactation stages (PE: Pre-experimental; EL: early lactation; ML: Mid-lactation; LL: Late lactation), the effect of the 3 diets tested (CD: control diet; BD: broccoli by-product; APD: artichoke plant by-product) and the refrigerated storage time of fermented milk (2 and 30 days) on the variables analyzed (microbial count, pH, fat, protein, total solids, lactose, glucose, galactose, fatty acids, lactic acid, syneresis, and, whey fat, protein, lactose and total solids). However, for the variables total polyphenol content and antioxidant capacity (ABTS and DPPH), only EL and LL productions were analyzed. For volatile profile only PE and LL were analyzed. The proc model GLM used was the following (Equation (15)):

*Y* = *μ* + *DIETi* +*TIMEj* + *DIETi* × *TIMEj* + *eijk* (15)

*Y* = dependent variable; *µ*: mean; *DIETi*: effect of diet (*n* = 3: control, broccoli by-product, and artichoke plant by-product); *TIMEj*: storage time of fermented milk (*n* = 2: 2 and 30 days); *DIETi* × *TIMEj*: interaction of diet and time; and, *eij*: residual error.

Principal component analysis (PCA regression map) was conducted to project the samples depending on the different chemical families of identified volatile compounds using XLSTAT Premium 2016 (Addingsoft, Barcelona, Spain).

## 3. Results and Discussion

### 3.1. Viability of Microbial Load and pH

**Figure 1:**
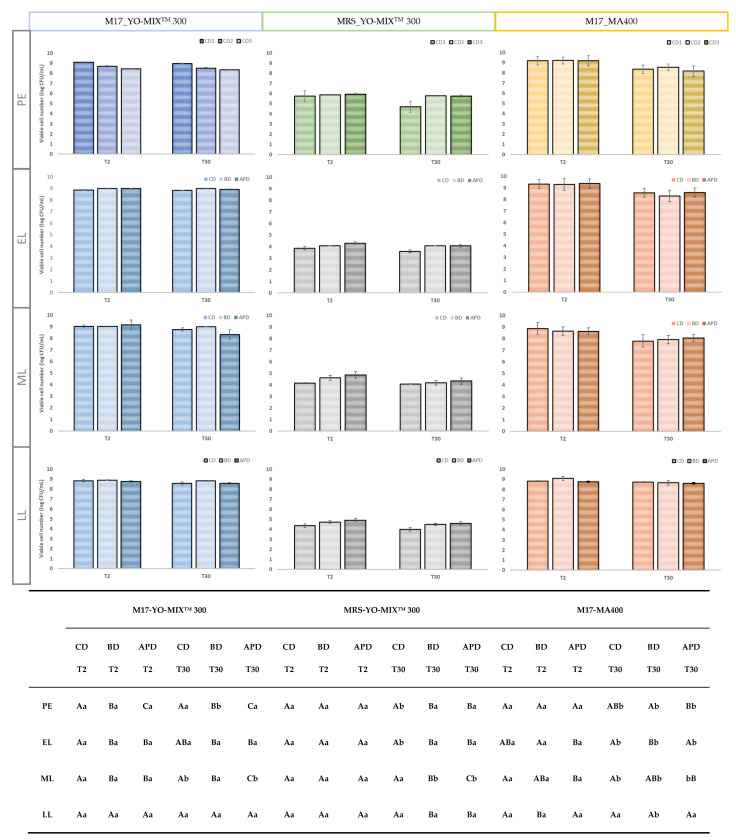
Lactic acid bacteria (viable cell number, log CFU/mL) of lactobacilli starter culture (YO-MIX^TM^ 300 in MRS agar) and lactococci and streptococci (YO-MIX^TM^ 300 and MA400 in M17 agar) in fermented milk with a storage time under refrigeration (T2 and T30). PE: Pre-experimental; EL: Early lactation; ML: Middle lactation; LL: Late lactation; CD1 Control diet; BD: Broccoli by-products diet; APD: Artichoke plant by-products diet. The lowercase letters refer to the differences between days (T2 and T30) of the same type of diet in each of the rows (production); Capital letters refer to the differences between diets at each of the times for each of the rows (production).

The survival of microorganisms during processing and storage in fermented milk is highly important [40]. As can be seen in Figure 1, the type of diet, storage time and lactation stage did not significantly affect the number of viable microorganisms of all milk fermented by YO-MIX^TM^ 300 starter culture. The sum of the number of viable cells of *Streptococcus thermophilus* and *Lactobacillus delbrueckii* spp. *bulgaricus* was >10^7^ CFU/mL (colony forming units CFU) and the ratio 2:1 (*S. thermophilus: L. bulgaricus*) was maintained during refrigerated storage, being the established requirement for yogurt fermented milk by CODEX ALIMENTARIUS [25,40,41,42]. The symbiosis between these microorganisms has been extensively studied, the protocooperation. Markakiou et al. (2020) [26] explained the sum of the acid production in a mixed culture is greater than in a single culture. *Streptococcus thermophillus* produce several components (formic acid, pyruvic acid, carbon dioxide, long chain fatty acids, among others) which stimulate the growth of *L. delbrueckii* spp. *bulgaricus*, which is highly proteolytic. This causes a pH reduction due to the transformation of lactose into lactic acid, and then, the denaturation of milk proteins and the release of aminoacids that are essential for the growth of *Streptococcus.* According to Liu et al. (2016) [13] the fermentation time decreases when symbiotic cultures are used. Previously, Dimitrellou et al. (2019) [25] found differences in the bacterial count of *S. thermophilus* and *L. delbrueckii* spp. *bulgaricus* in fermented goat milk, increasing the viability of *S. thermophilus* even under low temperature conditions and after 28 days of storage, while the viability of *L. delbrueckii* spp. *bulgaricus* decreased. In our study, a general reduction of viable cells was detected with the advance of the lactation stage; counts on FMs at PE and EL productions (three diets) were higher than those on FM from LL stage.

As to FMs by MA400 starter culture, a general decrease in the number of viable cells was observed due to the storage time from T2 to T30 of refrigeration (*p* < 0.05) for the three studied diets and at the four lactation stages (PE; EL; ML and LL). Considering lactation stage and diet, although some significant differences were detected they were considered irrelevant as they were minor in quantitative terms. No scientific studies on fermented milks by MA400 culture were found in the scientific literature as it is especially used for the manufacture of cheese, and also butter and buttermilk. The decrease in viability during storage cannot be directly attributed to any of the microorganisms that are part of this starter culture, but it could be potentially linked to *Lactococcus lactis. L. lactis* has been used together with other microorganisms in cheese making and it has been observed after six days of storage at 6 °C, that the decrease in the number of viable cells was due to *L. Lactis* reduction (from 2.3 × 10^9^ CFU/g to 1.7 × 10^9^ CFU/g) [43]. This decrease was attributed to the fact that while LAB grows, lactate is increasing which causes a reduction in pH, inhibiting the growth of *L. lactis* [44]

**Figure 2:**
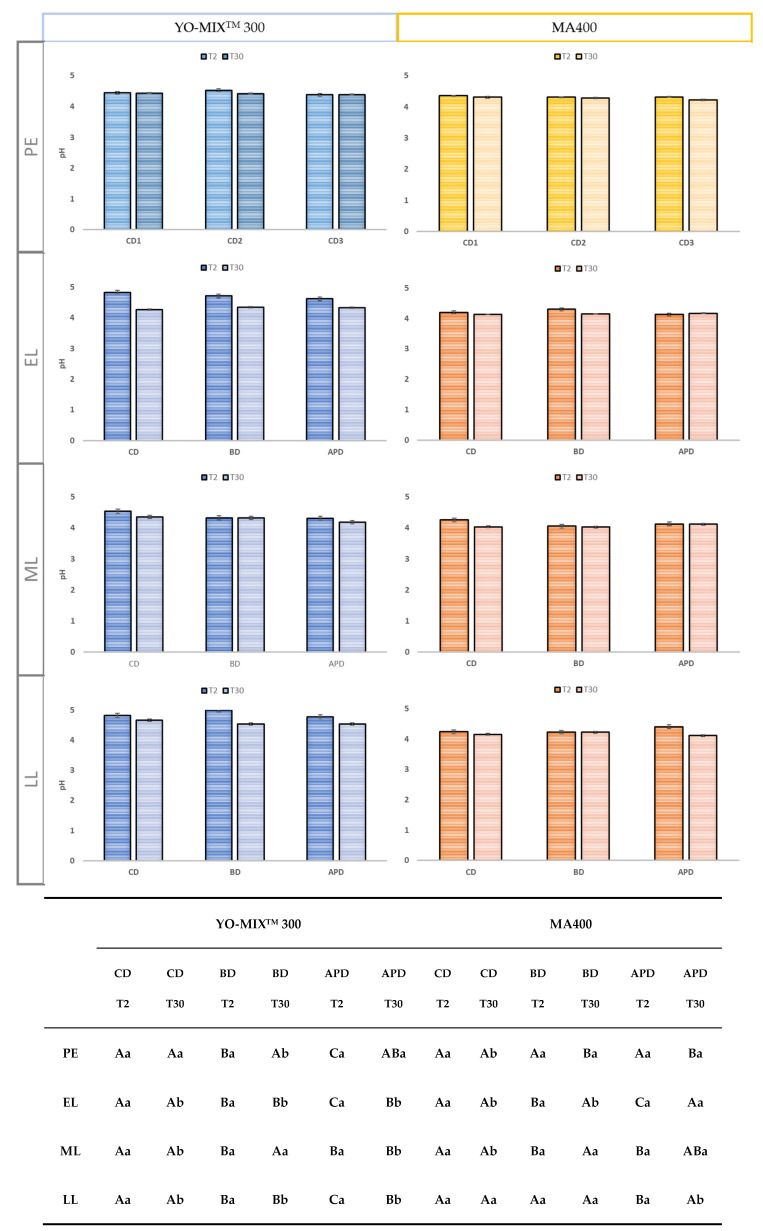
pH value of fermented milk during refrigerated 2 and 30 days at 4 °C using a thermophilic starter culture (YO-MIX^TM^ 300) and mesophilic starter culture (MA400). PE: Pre-experimental; EL: Early lactation; ML: Middle lactation; LL: Late lactation; CD: Control diet; BD: Broccoli by-products diet; APD: Artichoke plant by-products diet. The lowercase letters refer to the differences between days (T2 and T30) of the same type of diet in each of the rows (production); Capital letters refer to the differences between diets at each of the times for each of the rows (production).

One of the key parameters in FMs production is pH decrease in milk; it is the direct consequence of the activity of the inoculated microorganisms. Fast acidification ensures food safety and pH is used as an indicator of the end point of fermentation. No significant differences were found among diets and lactation stage (Figure 2). FMs by YO-MIX^TM^ 300 (range 4.6 and 4.18) had higher pH values than FMs by MA400 (range 4.36 and 4.02). A slight decrease of pH was noticed between T2 and T30 for both cultures. These results agree to what was previously found by other authors, were no significant variations of pH during storage for FMs was noticed. It is observed that the slight reduction of pH, especially FMs by YO-MIX^TM^ 300 and an increase in acidity could be explained by the improvement of microbial growth and the peptidase activity of *L. delbrueckii subsp. bulgaricus*, which is greatly favoured in goat milk [41,45]. Moreover, the urease activity of *S. thermophilus* present a significant correlation with the use of lactose and the production of lactic acid [24,27].

### 3.2. Composition of Milk and Fermented Milk

Composition of raw bulk tank milk is shown in Table 1. Regarding fat content, a 10% increase was observed at the EL stage sampling as compared to PE sampling for diets BD (5.30% vs. 4.80%) and APD (5.07% vs. 4.56%), the increase was only slight for group on CD diet (4.91% vs. 4.73%). Fat content increased till the end of lactation reaching values of 5.39% in CD and 5.61% in BD. However, fat content in APD remained constant throughout the experiment. BD diet yielded the milk with the highest fat content throughout the study. Protein content was slightly lower in BD and APD with respect to CD at EL and ML stages (0.1 units *p* < 0.05). An increase in protein was observed at the end of lactation (LL) for all diets, reaching contents of 3.62%, 3.56% and 3.42% for CD, BD and APD, respectively. Protein content was always higher in milk from the CD diet. A parallel study conducted by Monllor et al. [8] with an inclusion of 40% broccoli and artichoke plant by-products throughout lactation, observed similar results with an increase of 0.5% in absolute terms of fat in milk from of diets with broccoli silage and a higher protein content for milk from a conventional diet. They also observed an increase in fat and protein throughout lactation. Other parallel studies [6,7,8,29] but with a short-term inclusion showed that 40% broccoli silage by-products and 40% and 12.5% artichoke plant silage also increased the percentage of fat with respect to the conventional diet. However, the percentage of protein was hardly modified by the differentiated diets.

The average macrocomposition of the milk fermented by YOMIX^TM^ 300 starter culture is presented in Table 2. As to fat percentage, an increase throughout lactation was detected, being more pronounced in fermented milks from BD diet with an interval of 4.46–5.50% between PE and LL production, with an increase (*p* < 0.05) of 1.04 percentage units. Fermented milk derived from CD also showed the same tendency but slightly less pronounced with an increase of 0.52 percentage units between PE and LL production, followed by fermented milks made from APD (an increase of 0.43 percentage units). As to protein percentage, a similar trend was observed, increasing throughout lactation being LL production significantly higher than PE in all batches (0.38, 0.32 and 0.19 percentage units for CD, APD and BD, respectively). This increase was also observed ML stage in the CD batch. The tendency above described for protein content in milk with the same proportion in the goat diet (40% of by-product) was maintained when milk is fermented by YO-MIX^TM^ 300: CD (3.75%) presented higher values than APD (3.67%), followed by BD (3.54%). 

Regarding the composition of milk fermented by MA400 starter culture (Table 3) the same tendency was observed, increasing fat and protein content during lactation. A fat percentage increase (*p* < 0.05) of 0.24 percentage units was observed in the BD diet (5.63%) compared to the APD (5.41%) and CD (5.39%) diets between PE and LL. These differences were also found in milk from goat fed with the incorporation of 40% of by-product. Milk from animals fed broccoli by-product silage had a higher average fat content compared to the conventional diet and with artichoke plant by-product silage [6,8,29]. Nevertheless, when milk was fermented by MA400 no differences in protein percentage were found between APD and BD (3.45%).

Fermented milks macrocomposition (from both starter cultures) were not affected by the studied storage time. In some cases, significant differences were detected but these results presented no clear trend, being the differences between T2 and T30 very small (in percentage units). Average macrocomposition of the fermented milks with the different starter cultures and in the different productions and diets did not quantitatively differ much. Consequently, when evaluating gel stability (syneresis and whey composition) the behavior was similar, as will be discussed later. 

Table 4 and Table 5 show the sugars (lactose, glucose and galactose) and lactic acid present in the FMs. These components contribute to the characteristic taste of this type of product. Lactose content in raw milk ranged from 4.75 to 4.29%, and was reduced by fermentation with YO-MIX^TM^ 300 and MA400 to values up to 1.78 and 1.70%, and 1.72 and 1.56% after T2 and T30 during refrigerated storage, respectively. Significant differences were observed between T2 and T30 of storage in some elaborations (*p* < 0.05) but with no clear trend, and could be considered caused by the elaboration process. It can be said that the highest content of lactose was observed in PE and EL FMs and the lowest content in LL FMs. In previous studies [46] the residual lactose was 1.90% in FMs by *L. bulgaricus*, which is one of the YOMIX^TM^ 300 microorganisms in the present study. Data from Muelas et al., 2018 [46] agree with the observation that only traces of glucose were detected in EL in T2 with MA400 culture, but it was observed that glucose values increased (0.24 and 0.34% for T2 and T30 of refrigeration, respectively) in later lactations (EL and ML) and T30 of storage. A decrease of glucose in FMs at the end of lactation was noticed.

It is noteworthy that the highest values of galactose were reached in YO-MIX^TM^ 300 FMs, and regarding lactation stage PE had the highest values which were reduced throughout lactation. As to MA400 fermented milk, a slight increase of galactose values was observed during lactation, however residual levels of glucose and galactose were both in similar range and not clearly influenced by the factors under study storage time and feeding [46]. Although statistically differences were detected (*p* < 0.05), these may not be considered relevant.

The lactic acid content was not affected by storage time, whereas due to the diet quantitatively slight differences (*p* < 0.05) were detected but with no clear trend. Lactic acid content was consistent with pH values. YO-MIX^TM^ 300 FMs had less lactic acid and higher pH than those from MA400. Muelas et al., 2018 [46] reported a lactic acid content of 0.59% in FMs with MA400, similar to the present study in PE. It can be seen that it increases to higher than 0.70% from week 12 of lactation and it is maintained until the end of the experiment with slight variations. It was also observed that lactic acid content was higher in fermented milk from broccoli by-product feeding, which was also the one with the highest dry matter content.

### 3.3. Syneresis and Whey Composition

Syneresis in FMs is the separation of the liquid phase from the gel. According to Domagała (2009) [47] it can occur spontaneously or when the gel is mechanically disrupted during cutting, shaking or freezing, and is undesirable in firm and stirred fermented milk because it can negatively influence consumer acceptance of the food product. In previous studies, it has been observed that when the protein content and total solids are increased, an increase of whey retention capacity is observed, and then spontaneous syneresis process disappear, obtaining products with greater apparent viscosity and firmness [47,48]. Table 6 and Table 7 show the percentage of syneresis and composition of the expelled whey. It is observed that the syneresis values are slightly higher in FMs with YO-MIX^TM^ 300 compared to those obtained with MA400. Similar values (average 64%) were found in previous studies [46]. Other authors [48] compared the percentage of syneresis 14 days after fermentation in yogurts based on different types of milk, obtaining considerably higher values in goat milk (39%) compared to cow milk (25%) or sheep (17%). Martín-Diana et al. (2003) [41] explained that when goat milk is used for FMs, it is required the inclusion of a fortification to improve the coagulum characteristics because goat milk presents slightly lower casein content (α-s1-casein) than cow’s milk. The content of αs1-casein in goat milk depends on the genetic polymorphisms, whereas goats with alleles A, B or C have contents of αs1-casein up to 25% of the total milk protein, goats with O or N alleles have no αs1-casein. The lower the content of αs1-casein the larger the casein micelles and the number of hydrated pores yielding a less dense gel structure than cow milk [49]. It is important to mention that no significant differences on syneresis were observed between storage times, as opposed to observations by Domagała, (2009) [47] who did appreciate a decrease due to storage. Other authors also reported increased percentage of syneresis with storage time in cow’s milk yogurt [22]. In a previous study, whey composition was as follows: 0.32% protein, 0.24% fat, 4.4% lactose, and 3.5% of total solids [46] In the present study, lactose was much lower, presenting values <2.5%. It should be noted that the protein values were around 0.30% and decreased considerably in all fermented milks regardless of the feeding, at late lactation. It can be observed that FMs cultured with MA400 lost more fat with the whey than those cultured with YO-MIX^TM^ 300. 

### 3.4. Functionality of Fermented Milks

#### 3.4.1. Antioxidant Capacity and Total Phenols

At late lactation, no significant differences in TPC of raw milk were observed due to the diet (Table 8). Compared with raw milk, there was a slight increase in TPC after fermentation with both thermophilic and mesophilic cultures. It is important to highlight that the lowest TPC values of FMs at the end of lactation were found in the batch where the diet was conventional, while the highest values were obtained when 40% broccoli by-product was incorporated. It would be interesting to observe in future studies if there is an increase of other bioactive compounds such as sulfur compounds (glucosinolates and/or isothiocyanates such as sulforaphane). No differences between storage times (T2 and T30) were observed in this study, so only results from day 30 are presented in Table 8. Degradation of phenolic compounds (specially anthocyanins) in fermented milk was noticed during fermentation and storage in previous studies [22,50], however, in the present study no differences were detected between days 2 and 30 of storage. Regarding antioxidant activity, DPPH values did not show significant differences between diets in both raw milk and fermented milk, except when mesophilic culture was used. As to ABTS assay, there is a significant increase in raw milk when incorporating 40% artichoke by-product into the diet but no significant differences were observed between the different diets in fermented milks. Although it would need more research, this change in the antioxidant capacity from raw to fermented milk could be caused by the differences of available soluble peptides, which provide great antioxidant capacity (mainly ABTS) in milk and fermented milk [51].

#### 3.4.2. Fatty Acid Health/Nutritional Indexes

Fatty acid health/nutritional indexes of raw milk and fermented milk of late lactation stored 30 days (LL) are presented in Table 9. Late lactation sampling of 30 days stored FMs was the only sampling selected to be included in the table to avoid repetitive tables, and to represent the most extreme conditions (longest period under differentiated diets). At PE sampling milk from different batches did not differ, while differences due to diet were evident at EL, ML as well as the presented results of LL. No differences were detected between 2 and 30 days of refrigerated storage. 

Chen and Liu, 2020 [16] recently reviewed health/nutritional indexes in different foods, including dairy foods. All values in Table 8 are within the ranges reported for dairy goat products in the scientific literature for those indexes. PUFA/SFA is a general index for the nutritional value of fats and ranges from 0.02–0.175 in dairy goat products. Indexes n6/n3 and LA/ALA are a quality index for baby foods given the relevance on n3 fatty acids and ranges in cow’s milk are 2.46–3.44, no data was reported for goat milk. Aterogenicity index (IA) and thrombogenicity index (IT) in goat milk foods range 1.89–2.91 IA, 2.70–3.20 IT. Hipocholesterolemic/hypercholesterolemic index (HH) have no data reported in goat milk products, they range from 0.32 to 1.29 in other dairy foods. Health promoting index (HPI) is the inverse of IA and ranges from 0.37 to 0.68 in goat cheeses. The following are desaturase indexes that relate each unsaturated fatty acid to the previous saturated form ∑CLA/Vaccenic acid, DI14, DI16, and Oleic/estearic acid and DI18 which are closely related.

Milk fermentation by both cultures had little influence on the fatty acid profile and calculated indexes. Only n6/n3-LA/ALA together with hypercholestrolemic index (HFA) increased due to fermentation and all desaturase indexes decreased. Main modifications in health indexes are in fact due to the inclusion of silages from both broccoli and artichoke plant and they are discussed together. 

The incorporation of broccoli (BD) and artichoke by-products (APD) in the diet modified the fatty acid profile of raw milk (RM), and consequently all the calculated fatty acid ratios/indexes in the RM were modified, except DI16. DI16 is a desaturation index (DI14, DI16 and DI18), being this related to the isomers of C16:1 and C16:00. Previous studies [7,8,29] indicated that silages from APD included in dairy goat balanced diets up to a 12.5% (12.5% APD) and 25% (25% APD) replacement of conventional ingredients showed similar MUFA/SFA and PUFA/SFA values to CD values [6,7,29]). Although n6/n3 and IA values (11.03 and 2.41, respectively) were higher in 12.5% APD than CD (8.94 and 2.39, respectively), TI was higher in CD (3.14) than APD (3.08) [7]. When 25% APD was included, IA and TI were similar to CD (Monllor et al., 2021). When 40% APD and BD were included in the goat diet for a short period, similar values of MUFA/SFA and PUFA/SFA were found in 40% APD and CD (mean values 0.40 and 0.07, respectively), followed by BD with lower values (0.37 and 0.05, respectively) [52]. The tendency observed in previous studies related to n6/n3 values when <40% APD was not in agreement with our study in which 40% APD was included. N6/n3 values were higher in CD (14.66) than BD (13.35), followed by APD (11.93) [7] In the present study, slight reductions of MUFA/SFA, Hypocholesterolemic/Hypercholesterolemic ratio (HH), ∑CLA/Vaccenic acid, atherogenicity index (IA), and health promoting index (HPI) of RM were observed when 40% APD and 40% BD were added on the diet. Reductions of n6/n3, and LA/ALA were highly relevant. Although all observed values are within values previously reported in the scientific literature, it can be also said that when 40% BD and APD are included in the diet a slight increase of hypercholesterolemic index (HFA) of raw milk was observed. Thrombogenicity index (IT) was slightly increased as well, when 40% BD was included in the diet, while when 40% APD was added, no effect was observed. It is important to highlight the increase of oleic/estearic, DI14 and DI18 in both raw and fermented milk when 40% APD was included.

Table 9 shows that IA presented statistically higher values in FMs by MA400 than by YO-MIX^TM^ 300. ∑CLA/Vaccenic acid was increased in raw milk when by-products were included in the diet, but this statistical changes disappear when milk was fermented. Previous studies detected a lower concentration of linoleic, vaccenic and rumenic acids in milks when broccoli by-product was used as feeding due to the lower proportion of linoleic and α-linolenic in broccoli stalks and leaves, precursors of aforementioned acids [8].

Taking the enrichment of formulation with by-products into account, although all observed values are in the common range for goat milk products, and better than those reported for cows’ milk, it can be said that when 40% BD and APD, a slight increase of hypercholesterolemic index (HFA) of FMs by both YO-MIX^TM^ 300 and MA400 cultures were observed due to the increase of total C12:00 + C14:00 + C16:00. Thrombogenicity index (IT) was significantly increased when 40% BD was included in the diet in FMs by both studied cultures, whereas when 40% APD was added, no effect was observed. An increment of IA was noted when feeding was fortified with 40% BD when FMs were obtained with YO-MIX^TM^ 300 culture. Related to FMs by both cultures, MUFA/SFA, LA/ALA, and health-promoting index (HPI) decreased due to fermentation. In the case of PUFA/SFA the values were decreased when 40% BD was used, while no effect was detected when 40% APD was included during feeding. It is important to highlight the increase of oleic/estearic, DI14 and DI18 in fermented milk when 40% APD was incorporated into the diet. The mentioned health indexes (oleic/estearic, DI14 and DI18) were not affected when 40% BD was incorporated into the diet. 

To summarize this section, it can be mentioned that the inclusion of broccoli and artichoke by-products slightly affected health/nutritional indexes, being values from artichoke diet similar to those of control diet or enhanced (increased oleic acid and desaturase indexes). All calculated indexes were within the ranges reported for goat’s milk products and better than those of most cows’ milk products. Depending on which kind of by-product was added during feeding, the effect was different. While the fortification of BD slightly increased IA and IT, the incorporation of APD did not modify the mentioned ones, and enhanced ∑CLA/Vaccenic acid and other desaturase indexes. 

### 3.5. Volatile Profile of Fermented Milks

Main volatile compounds in fermented milks were analyzed in samples from PE (before introducing silages in the diet) and LL (after four months in different diets), results are presented in Table 10 as prevalence of each volatile compound (% of area). Fifty-one compounds were identified in milks fermented by the mesophilic culture MA400 being 11 aldehydes, 8 hydrocarbons, 8 terpenes, 7 ketones, 6 esters, 5 alcohols, 5 acids and 1 sulfur compound. Forty-five compounds were identified in milks fermented by the thermophilic culture YO-MIX^TM^300: 10 aldehydes, 7 hydrocarbons, 6 terpenes (some of them in the limit below 0.2 prevalence), 7 ketones, 6 esters, 4 alcohols, 4 acids and 1 sulfur compound. The prevalence of the chemical families is similar to the previously reported in other fermented goat milk products [19]. Most of the compounds were isolated in both type of fermented milks, although with different prevalence. Major compounds in milk cultured with both cultures were hexanal, 2-heptanone, heptanal, hexanoic acid, octanal, 2-ethylhexanol, benzyl alcohol, 2-nonanone, nonanal and nonanedienol.

Five of the identified compounds are considered key compounds in the flavor of fermented milks: acetoin, hexanal, 2-heptanone, 2-nonanone and nonanal [53]. Regarding the compounds mainly responsible for the goaty flavor, hexanoic acid was the most prevalent and showed a tendency to increase at late lactation and also in fermented milk from broccoli BD and artichoke plant silages APD fed groups. Hexanoic acid is also recognized as a major source of flavor in fermented milks [54]. Octanoic and decenoic acids had a lower prevalence and were not affected by lactation stage.

The prevalence of volatile compounds in fermented milks by mesophilic culture was modified due to lactation stage for the following compounds: hexanal, heptanal, dodecane and tridecane decreased at late lactation, whereas butanoic acid, 2-heptanone, 2-nonanone increased. Regarding the diet, it significantly modified the prevalence of several compounds. Butanoic acid, hexanoic acid, octanal and octanoic acid were more prevalent in those FMs from diets including silages (BD and ADP), whereas heptanal, benzyl alcohol, nonanal and methyl salicylate contents were lower than in CD. The inclusion of broccoli significantly (*p* < 0.05) increased 2-heptanone prevalence in fermented milk. In the case of the thermophilic culture, the lactation stage affected acetoin, hexanal, heptanal, octanal, nonadienal and decenal that decreased, whereas 2-heptanone, hexanoic acid, ethyl-hexenol, benzyl alcohol, 2-nonanone, octanoic acid and decanal increased at late lactation. The diet caused significant differences on hexanal, nonanal and decanal that increased due to the inclusion of silages (BD and APD) and 2-heptanone and octanoic acid that decreased as compared to CD at late lactation. The inclusion of broccoli silage yielded milk with reduced prevalence of heptanal and cubebene. The inclusion of artichoke plant silage caused a decreased prevalence of 2-nonanone. Overall, aldehydes decreased at late lactation in both fermented milks regardless the diet. Aldehyde content in fermented milks is mainly dependent on the enzymatic activity of the substrate as they are produced from the degradation of milkfat or from the catabolism of aminoacids, and they are degraded by oxidation to carboxylic acids or by reduction to alcohols [55]. In milk cultured with MA400 the prevalence of acids increased when silages were included in the diet, so expecting those products as having higher goaty flavor, whereas in milk fermented with yogurt culture aldehydes prevalence increased when silages were included, so expecting green and fresh flavors. When handling the samples, authors perceived them all as having mild odor and no evident odor differences were detected among groups, in the future, sensory analysis would be of interest to evaluate flavor perception in detail.

**Figure 3:**
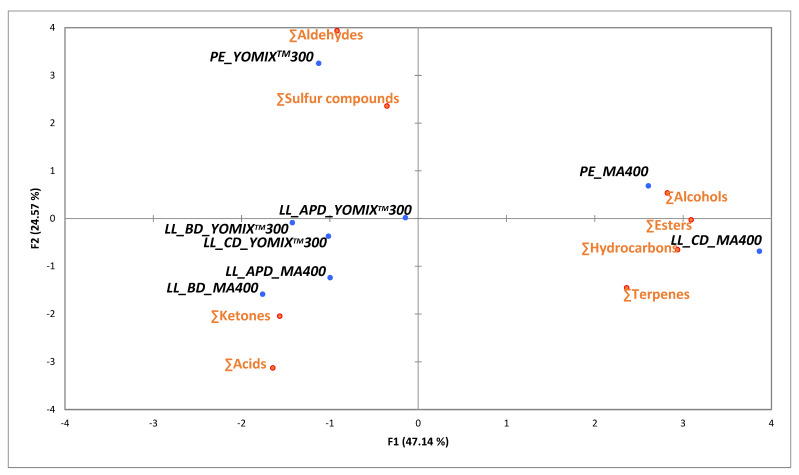
PCA scores plot showing the relationship among different chemical families of volatile compounds in fermented milks at pre-experimental stage (PE) and late lactation stage (LL_CD: conventional fed; LL_BD: broccoli fed; and, LL_APD: artichoke plant fed) by MA400 and YO-MIX^TM^ 300.

For an easy visualization of the relationships among volatile compounds, a PCA was run, including only significantly different chemical families of volatile compounds. Figure 3 shows the two principal components which explained 71.71% of the samples variation. Milks fermented by YO-MIX^TM^300 were closely located and positively correlated with aldehydes prevalence, whereas in milk fermented by MA400 the inclusion of silages positively correlate with the prevalence of acids and were clearly separated from CD and PE fermented milks.

Although diets are expected to greatly affect the aroma of milk, the fact that so little significant differences in volatiles could be linked to the different diets may be related to the previously reported observation that flavor complexity is highest when animals are fed a mixed ration including concentrates [56] as all diets included concentrates, besides, lactic acid culture is considered the main factor affecting the volatile profile of fermented milks [54].

From all the identified families of compounds terpenes have been proposed as indicators of the diet mainly when animals were grazing different pastures and their content may by five times higher when in pasture as compared to in-door feeding [57]. In fact, they have been proposed as indicators of mountain pasture milk [58], some others reported that even in semi-pasture combined with in-door feeding terpenes do not allow the differentiation with in-door feeding [59]. Regarding goat milk, it has been pointed out that terpenes may not be a good indicator of the diet [60]. In the present study the feeding system was in-doors and included silages. Fan et al. (2020) reported that terpenoid from artichoke were well preserved in silages preserved for 60 days, and main changes in their profile occurred during the first days of silage and were highly dependent on the microbiota in the silage [61]. In the present study silages had been stored for much longer times and no presence of the terpenoids reported in artichoke [61] were found in fermented milks. Terpenes may suffer further transformations, they may be directly transferred from the diet (on inhaled) to milk or may be further modified in the rumen (biohydrogenated and isomerized) [62]. Even during milk fermentation terpenes may undergo further modifications [57].

## 4. Conclusions

Long term inclusion of 40% silage from broccoli and artichoke plant by-products in balanced diets of dairy goats yields milk suitable for yogurt and cheese fermentation. Broccoli inclusion enhances fat and solids content in milk and consequently in fermented milks, artichoke plant inclusion does not pose differences as compared to milk from conventional diets. Gel stability, lactic acid bacteria counts, fermentation performance (acidity and organic acids and sugars profile) are not affected by diets. The inclusion of broccoli silage enhances antioxidant properties of milk. Fermentation enhances antioxidant properties of fermented milks from all diets, especially when mesophilic cheese starter cultures are used. Artichoke plant silage inclusion does not modify antioxidant properties as compared to conventional diets. Health quality index of milk from conventional and silage fed dairy goats are within usual values reported for goat milk products, whereas artichoke plant inclusion improves health indexes due to enhanced unsaturated fatty acids profile. Volatile aldehydes in fermented milks decrease with the advance of lactation. The inclusion of both silages enhances hexanoic acid and aldehyde contents in yogurt cultured milk and enhances butanoic, hexanoic and octanoic acids as well as octanal in milk fermented by the mesophilic culture. Volatile terpenes do not allow to differentiate among the studied diets. Differences on the prevalence of volatiles point to moderate to low differences on the flavor of fermented milks, however further studies are needed including sensory analysis to provide a deeper inside on the effect of those diets on milk flavor. The inclusion of silages from broccoli and artichoke plant by-products in the diet of dairy goats represents a truly implementation of a sustainable farming practice in line with the present trends towards circular economy strategies. Further studies are needed to evaluate the incorporation of other byproducts in the long term on the diet of dairy ruminants and further check milk suitability for industrialization to contribute to the sustainability of farming.

## Figures and Tables

**Table 1:** Principal components (fat, protein, lactose and total solids) content (g/100 mL) in raw milk used for the different productions of fermented milk.

Production	Diet	Fat	Protein	Lactose	Total Solids
PE	CD1	4.73 ± 0.02 A	3.37 ± 0.01 A	4.73 ± 0.02 A	13.33 ± 0.01 A
	CD2	4.80 ± 0.02 B	3.35 ± 0.01 A	4.73 ± 0.02 A	13.31 ± 0.01 B
	CD3	4.56 ± 0.02 C	3.34 ± 0.01 A	4.74 ± 0.03 A	13.10 ± 0.01 C
	Anova	**	NS	NS	**
EL	CD	5.12 ± 0.02 A	3.34 ± 0.02 A	4.57 ± 0.01 A	13.50 ± 0.10 A
	BD	5.30 ± 0.02 B	3.21 ± 0.02 B	4.59 ± 0.01 A	14.00 ± 0.10 B
	APD	5.07 ± 0.02 C	3.26 ± 0.02 B	4.58 ± 0.01 A	13.27 ± 0.10 C
	Anova	*	*	NS	*
ML	CD	5.15 ± 0.02 A	3.41 ± 0.01 A	4.47 ± 0.11 A	14.33 ± 0.02 A
	BD	5.56 ± 0.02 B	3.29 ± 0.01 B	4.52 ± 0.11 A	14.50 ± 0.02 B
	APD	5.04 ± 0.02 C	3.28 ± 0.01 B	4.47 ± 0.11 A	13.94 ± 0.02 C
	Anova	***	*	NS	*
LL	CD	5.39 ± 0.05 A	3.62 ± 0.02 A	4.35 ± 0.03 AB	13.90 ± 0.05 A
	BD	5.61 ± 0.05 B	3.56 ± 0.02 A	4.39 ± 0.03 A	14.18 ± 0.05 B
	APD	5.10 ± 0.05 C	3.42 ± 0.02 B	4.29 ± 0.03 B	13.21 ± 0.05 C
	Anova	*	**	*	***

PE: Pre-experimental; EL: Early lactation; ML: Middle lactation; LL: Late lactation; CD1: Control diet; BD: Broccoli by-products diet; APD: Artichoke plant by-products diet. Least square means within a column having different letters differ significantly. * *p* < 0.05; ** *p* < 0.01; *** *p* < 0.001. NS: not significant. Capital letters refer to the differences between diets at each of the times for each of the rows (production).

**Table 2:** Main components (fat, protein and total solids) content (g/100 g) in fermented milk manufactured and stored for 2 and 30 days with milk from Murciana-Granadinas goats fed differentiated diets, using a thermophilic (YO-MIX^TM^ 300) starter culture.

Production/Diet		Fat			Protein			Total Solids		
		T2	T30	Anova	T2	T30	Anova	T2	T30	Anova
PE	CD1	4.77 ± 0.02 Aa	4.86 ± 0.02 Ab	*	3.37 ± 0.01 Aa	3.35 ± 0.01 Aa	NS	13.16 ± 0.01 Aa	13.26 ± 0.01 Ab	***
	CD2	4.66 ± 0.02 Ba	4.74 ± 0.02 Bb	*	3.34 ± 0.01 Aa	3.35 ± 0.01 Aa	NS	13.09 ± 0.01 Ba	13.05 ± 0.01 Ba	NS
	CD3	4.35 ± 0.02 Ca	4.37 ± 0.02 Ca	NS	3.35 ± 0.01 Aa	3.35 ± 0.01 Aa	NS	12.73 ± 0.01 Ca	12.72 ± 0.01 Ca	NS
	Anova	**	**		NS	NS		**	***	
EL	CD	4.96 ± 0.13 Aa	5.03 ± 0.13 Aa	NS	3.34 ± 0.02 Aba	3.35 ± 0.02 Aa	NS	13.96 ± 0.10 Aa	13.26 ± 0.10 Ab	**
	BD	5.17 ± 0.13 Aa	5.19 ± 0.13 Ba	NS	3.35 ± 0.02 Aa	3.24 ± 0.02 Bb	*	14.10 ± 0.10 Aa	14.24 ± 0.10 Ba	NS
	APD	4.79 ± 0.13 Aa	4.42 ± 0.13 Ca	NS	3.24 ± 0.02 Ba	3.26 ± 0.02 ABa	NS	13.30 ± 0.10 Ba	13.47 ± 0.10 Aa	NS
	Anova	Ns	*		*	*		**	***	
ML	CD	5.13 ± 0.01 Aa	5.15 ± 0.01 Aa	NS	3.48 ± 0.01 Aa	3.44 ± 0.01 Aa	NS	14.12 ± 0.02 Aa	14..15 ± 0.02 Aa	NS
	BD	5.54 ± 0.01 Ba	5.51 ± 0.01 Ba	NS	3.33 ± 0.01 Ba	3.44 ± 0.01 Ab	**	14.46 ± 0.02 Ba	14.55 ± 0.02 Bb	*
	APD	4.98 ± 0.01 Ca	5.03 ± 0.01 Ca	NS	3.29 ± 0.01 Ba	3.25 ± 0.01 Ba	NS	13.77 ± 0.02 Ca	13.79 ± 0.02 CA	NS
	Anova	***	**		***	**		***	***	
LL	CD	5.29 ± 0.05 Aa	4.99 ± 0.05 Ab	**	3.75 ± 0.24 Aba	3.54 ± 0.24 Aa	NS	14.35 ± 0.05 Aa	14.14 ± 0.05 Ab	*
	BD	5.50 ± 0.05 Ba	5.23 ± 0.05 Bb	**	3.54 ± 0.24 Aa	3.34 ± 0.24 Aa	NS	14.46 ± 0.05 Aa	14.16 ± 0.05 Ab	*
	APD	4.78 ± 0.05 Ca	4.82 ± 0.05 Aa	NS	3.67 ± 0.24 Aa	3.45 ± 0.24 Aa	NS	13.47 ± 0.05 Ba	13.35 ± 0.05 Ba	NS
	Anova	*	*		NS	NS		***	***	

PE: Pre-experimental; EL: Early lactation; ML: Middle lactation; LL: Late lactation; CD1: Control diet; BD: Broccoli by-products diet; APD: Artichoke plant by-products diet. Least square means within a column having different letters differ significantly. * *p* < 0.05; ** *p* < 0.01; *** *p* < 0.001. NS: not significant. The lowercase letters refer to the differences between days (T2 and T30) of the same type of diet in each of the rows (production); Capital letters refer to the differences between diets at each of the times for each of the rows (production).

**Table 3:** Main components (fat, protein and total solids) content (g/100 g) in fermented milk manufactured and stored for 2 and 30 days with milk from Murciana-Granadinas goats fed differentiated diets, using a mesophilic (MA400) starter culture.

Production/Diet		Fat			Protein			Total Solids		
		T2	T30	Anova	T2	T30	Anova	T2	T30	Anova
PE	CD1	4.75 ± 0.02 Aa	4.86 ± 0.02 Ab	**	3.38 ± 0.02 Aa	3.36 ± 0.02 Aa	NS	12.75 ± 0.08 Aa	13.16 ± 0.08 Ab	**
	CD2	5.03 ± 0.02 Ba	4.97 ± 0.02 Ba	NS	3.36 ± 0.02 Aa	3.36 ± 0.02 Aa	NS	12.96 ± 0.08 Aa	13.33 ± 0.08 Ab	*
	CD3	4.85 ± 0.02 Ca	4.96 ± 0.02 Bb	**	3.35 ± 0.02 Aa	3.35 ± 0.02 Aa	NS	12.75 ± 0.08 Aa	13.33 ± 0.08 Ab	**
	Anova	*	*		NS	NS		NS	NS	
EL	CD	4.93 ± 0.02 Aa	4.95 ± 0.02 Aa	NS	3.36 ± 0.02 Aa	3.51 ± 0.02 Ab	***	13.43 ± 0.02 Aa	13.89 ± 0.02 Ab	***
	BD	5.54 ± 0.02 Ba	5.64 ± 0.02 Bb	***	3.14 ± 0.02 Ba	3.25 ± 0.02 Bb	**	13.85 ± 0.02 Ba	14.34 ± 0.02 Bb	***
	APD	4.92 ± 0.02 Aa	4.93 ± 0.02 Aa	NS	3.26 ± 0.02 Ca	3.35 ± 0.02 Cb	**	13.23 ± 0.02 Ca	13.47 ± 0.02 Cb	***
	Anova	**	*		**	*		***	***	
ML	CD	5.16 ± 0.02 Aa	5.44 ± 0.02 Ab	***	3.50 ± 0.02 Aa	3.43 ± 0.02 Ab	**	14.16 ± 0.02 Aa	13.94 ± 0.02 Ab	***
	BD	5.47 ± 0.02 Ba	5.75 ± 0.02 Bb	***	3.34 ± 0.02 Ba	3.25 ± 0.02 Bb	**	14.34 ± 0.02 Ba	14.02 ± 0.02 Bb	***
	APD	4.92 ± 0.02 Ca	5.22 ± 0.02 Cb	***	3.30 ± 0.02 Ba	3.26 ± 0.02 Ba	NS	13.75 ± 0.02 Ca	13.90 ± 0.02 Cb	***
	Anova	***	***		***	***		***	**	
LL	CD	5.39 ± 0.05 Aa	5.55 ± 0.05 Aa	NS	3.55 ± 0.02 Aa	3.62 ± 0.02 Aa	NS	13.39 ± 0.17 Aa	13.64 ± 0.17 Aa	NS
	BD	5.63 ± 0.05 Ba	5.89 ± 0.05 Bb	**	3.45 ± 0.02 Ba	3.55 ± 0.02 Ab	*	14.63 ± 0.17 Ba	14.55 ± 0.17 Bb	NS
	APD	5.41 ± 0.05 Aa	5.13 ± 0.05 Cb	**	3.43 ± 0.02 Ba	3.36 ± 0.02 Bb	*	13.09 ± 0.17 Aa	12.85 ± 0.17 Ca	NS
	Anova	*	**		*	***		***	*	

PE: Pre-experimental; EL: Early lactation; ML: Middle lactation; LL: Late lactation; CD1: Control diet; BD: Broccoli by-products diet; APD: Artichoke plant by-products diet. Least square means within a column having different letters differ significantly. * *p* < 0.05; ** *p* < 0.01; *** *p* < 0.001. NS: not significant. The lowercase letters refer to the differences between days (T2 and T30) of the same type of diet in each of the rows (production); Capital letters refer to the differences between diets at each of the times for each of the rows (production).

**Table 4:** Sugars and lactic acid content in fermented milk manufactured and stored for 2 and 30 days with milk from Murciana-Granadinas goats fed differentiated diets, using a thermophilic (YO-MIX^TM^ 300) starter culture.

Production/Diet		Lactose			Glucose			Galactose			Lactic Acid		
		T2	T30	Anova	T2	T30	Anova	T2	T30	Anova	T2	T30	Anova
PE	CD1	1.98 ± 0.02 Aa	2.01 ± 0.02 Aa	NS	0.39 ± 0.004 Aa	0.38 ± 0.004 Aa	NS	0.43 ± 0.006 Aa	0.45 ± 0.006 Ab	*	0.61 ± 0.005 Aa	0.62 ± 0.005 Aa	NS
	CD2	2.02 ± 0.02 Aa	2.02 ± 0.02 Aa	NS	0.39 ± 0.004 Aa	0.39 ± 0.004 Aa	NS	0.42 ± 0.006 Aa	0.42 ± 0.006 Ba	NS	0.57 ± 0.005 Ba	0.60 ± 0.005 Bb	NS
	CD3	2.00 ± 0.02 Aa	2.01 ± 0.02 Aa	NS	0.38 ± 0.004 Aa	0.39 ± 0.004 Aa	NS	0.42 ± 0.006 Aa	0.43 ± 0.006 Ba	NS	0.58 ± 0.005 Ba	0.59 ± 0.005 Ba	NS
	Anova	NS	NS		NS	NS		NS	*		**	*	
EL	CD	1.93 ± 0.03 Aa	2.01 ± 0.03 Aa	NS	0.39 ± 0.004 Aa	0.38 ± 0.004 Aa	NS	0.43 ± 0.05 Aa	0.42 ± 0.05 Aa	NS	0.57 ± 0.01 Aa	0.55 ± 0.01 Aa	NS
	BD	1.97 ± 0.03 Aa	1.88 ± 0.03 Ba	NS	0.39 ± 0.004 Ba	0.40 ± 0.004 Ba	NS	0.43 ± 0.05 Aa	0.42 ± 0.05 Ba	NS	0.61 ± 0.01 Aa	0.59 ± 0.01 Aa	NS
	APD	1.87 ± 0.03 ABa	2.09 ± 0.03 Aa	NS	0.38 ± 0.004 Ba	0.37 ± 0.004 Aa	NS	0.41 ± 0.05 Aa	0.40 ± 0.05 Aa	NS	0.60 ± 0.01 Aa	0.59 ± 0.01 Aa	NS
	Anova	*	*		**	***		NS	*		NS	NS	
ML	CD	1.89 ± 0.11 Aa	1.88 ± 0.11 Aa	NS	0.41 ± 0.01 Aa	0.39 ± 0.01 Aa	NS	0.49 ± 0.02 Aa	0.47 ± 0.02 Aa	NS	0.62 ± 0.01 Aa	0.62 ± 0.01 Aa	NS
	BD	1.96 ± 0.11 Aa	2.17 ± 0.11 Aa	NS	0.41 ± 0.01 Aa	0.38 ± 0.01 Aa	NS	0.44 ± 0.02 Aa	0.47 ± 0.02 Aa	NS	0.60 ± 0.01 Aa	0.62 ± 0.01 Aa	NS
	APD	1.88 ± 0.11 Aa	2.05 ± 0.11 Aa	NS	0.41 ± 0.01 Aa	0.34 ± 0.01 Ab	*	0.49 ± 0.02 Aa	0.50 ± 0.02 Aa	NS	0.61 ± 0.01 Aa	0.65 ± 0.01 Aa	NS
	Anova	NS	NS		NS	NS		NS	NS		NS	NS	
LL	CD	1.87 ± 0.02 Aa	1.72 ± 0.02 Ab	**	00.35 ± 0.05 Aa	0.36 ± 0.05 Aa	NS	0.40 ± 0.007 Aa	0.45 ± 0.007 Ab	**	0.50 ± 0.01 Aa	0.59 ± 0.01 Ab	**
	BD	1.93 ± 0.02 Aa	1.87 ± 0.02 Ba	NS	0.33 ± 0.05 Aa	0.35 ± 0.05 Aa	NS	0.38 ± 0.007 Aa	0.43 ± 0.007 Ab	**	0.52 ± 0.01 Aa	0.59 ± 0.01 Ab	***
	APD	1.78 ± 0.02 Ba	1.70 ± 0.02 Aa	NS	0.34 ± 0.05 Aa	0.35 ± 0.05 Aa	NS	0.39 ± 0.007 Aa	0.42 ± 0.007 Bb	*	0.50 ± 0.01 Aa	0.55 ± 0.01 Bb	**
	Anova	*	**		NS	NS		NS	*		NS	*	

PE: Pre-experimental; EL: Early lactation; ML: Middle lactation; LL: Late lactation; CD: Control diet; BD: Broccoli by-products diet; APD: Artichoke plant by-products diet. Least square means within a column having different letters differ significantly. * *p* < 0.05; ** *p* < 0.01; *** *p* < 0.001. NS: not significant. The lowercase letters refer to the differences between days (T2 and T30) of the same type of diet in each of the rows (production); Capital letters refer to the differences between diets at each of the times for each of the rows (production).

**Table 5:** Sugars and lactic acid content in fermented milk manufactured and stored for 2 and 30 days with milk from Murciana-Granadinas goats fed differentiated diets, using a mesophilic (MA400) starter culture.

Production/Diet		Lactose			Glucose			Galactose			Lactic Acid		
		T2	T30	Anova	T2	T30	Anova	T2	T30	Anova	T2	T30	Anova
PE	CD1	2.34 ± 0.02 Aa	2.60 ± 0.02 Ab	***	0.30 ± 0.002 Aa	0.33 ± 0.002 Ab	***	0.30 ± 006 Aa	0.31 ± 006 Aa	NS	0.61 ± 0.003 Aa	0.61 ± 0.003 Aa	NS
	CD2	2.28 ± 0.02 Ba	2.31 ± 0.02 Ba	NS	0.31 ± 0.002 Aa	0.34 ± 0.002 Bb	***	0.28 ± 006 Aa	0.30 ± 006 Ba	NS	0.59 ± 0.003 Ba	0.61 ± 0.003 Ab	NS
	CD3	2.28 ± 0.02 Ba	2.31 ± 0.02 Ba	NS	0.31 ± 0.002 Aa	0.34 ± 0.002 Bb	***	0.32 ± 006 ABa	0.33 ± 006 Ba	NS	0.59 ± 0.003 Ba	0.60 ± 0.003 Aa	NS
	Anova	*	***		NS	**		*	***		NS	NS	
EL	CD	2.14 ± 0.01 Aa	2.11 ± 0.01 Aa	NS	0.29 ± 0.001 Aa	0.28 ± 0.001 Aa	NS	0.28 ± 0.007 Aa	0.29 ± 0.007 Aa	NS	0.71 ± 0.006 Aa	0.75 ± 0.006 Ab	**
	BD	2.13 ± 0.01 Aa	2.09 ± 0.01 Ab	**	0.30 ± 0.001 Aa	0.29 ± 0.001 Bb	***	0.24 ± 0.007 Ba	0.25 ± 0.007 Ba	NS	0.75 ± 0.006 Ba	0.75 ± 0.006 Aa	NS
	APD	2.02 ± 0.01 Ba	2.05 ± 0.01 Bb	*	0.29 ± 0.001 Aa	0.27 ± 0.001 ABb	***	0.27 ± 0.007 Ca	0.27 ± 0.007 Ba	NS	0.72 ± 0.006 Aa	0.74 ± 0.006 Ab	***
	Anova	***	*		ns	***		*	***		*	ns	
ML	CD	1.85 ± 0.08 Aa	1.79 ± 0.08 Ab	***	0.30 ± 0.005 Aa	0.31 ± 0.005 Aa	NS	0.37 ± 0.009 Aa	0.39 ± 0.009 Aa	NS	0.61 ± 0.01 Aa	0.60 ± 0.01 Aa	NS
	BD	2.20 ± 0.08 Ba	1.78 ± 0.08 Ab	**	0.28 ± 0.005 Ba	0.32 ± 0.005 Ab	***	0.45 ± 0.009 Ba	0.39 ± 0.009 Ab	***	0.73 ± 0.01 Ba	0.67 ± 0.01 Bb	**
	APD	2.05 ± 0.08 ABa	1.80 ± 0.08 Aa	NS	0.29 ± 0.005 ABa	0.33 ± 0.005 ABb	***	0.40 ± 0.009 Ca	0.39 ± 0.009 Aa	NS	0.64 ± 0.01 Aa	0.62 ± 0.01 Aa	NS
	Anova	*	NS		*	*		*	NS		***	*	
LL	CD	1.80 ± 0.05 Aa	1.87 ± 0.05 Aa	NS	0.31 ± 0.007 Aa	0.24 ± 0.007 Ab	***	0.41 ± 0.007 Aa	0.31 ± 0.007 Aa	NS	0.67 ± 0.01 Aa	0.76 ± 0.01 Ab	***
	BD	1.84 ± 0.05 Aa	1.58 ± 0.05 Bb	**	0.31 ± 0.007 Aa	0.25 ± 0.007 Ab	***	0.40 ± 0.007 Aa	0.38 ± 0.007 Ba	NS	0.68 ± 0.01 Aa	0.70 ± 0.01 Bb	***
	APD	1.72 ± 0.05 Aa	1.76 ± 0.05 Aa	NS	0.30 ± 0.007 Aa	0.23 ± 0.007 Ab	***	0.40 ± 0.007 Aa	0.41 ± 0.007 Ab	*	0.66 ± 0.01 Aa	0.73 ± 0.01 ABb	**
	Anova	NS	*		NS	NS		NS	**		NS	**	

PE: Pre-experimental; EL: Early lactation; ML: Middle lactation; LL: Late lactation; CD1: Control diet; BD: Broccoli by-products diet; APD: Artichoke plant by-products diet. Least square means within a column having different letters differ significantly. * *p* < 0.05; ** *p* < 0.01; *** *p* < 0.001. NS: not significant. The lowercase letters refer to the differences between days (T2 and T30) of the same type of diet in each of the rows (production); Capital letters refer to the differences between diets at each of the times for each of the rows (production).

**Table 6:** Syneresis and main components (g/100 mL) of the expelled whey from fermented milk refrigerated at 4 °C during 2 and 30 days using a thermophilic starter culture (YO-MIX^TM^ 300).

Production/Diet		Syneresis			Fat			Protein			Lactose			Total Solids		
		T2	T30	Anova	T2	T30	Anova	T2	T30	Anova	T2	T30	Anova	T2	T30	Anova
PE	CD1	66.20 ± 1.06 Aa	65.74 ± 1.06 Aa	NS	0.35 ± 0.01 Aa	0.35 ± 0.01 Aa	NS	0.25 ± 0.01 Aa	0.26 ± 0.01 Aa	NS	2.28 ± 0.01 Aa	2.32 ± 0.01 Aa	NS	3.53 ± 0.01 Aa	3.52 ± 0.01 Aa	NS
	CD2	64.18 ± 1.06 Aa	63.13 ± 1.06 Ba	NS	0.35 ± 0.01 Aa	0.37 ± 0.01 Aa	NS	0.25 ± 0.01 Aa	0.26 ± 0.01 Aa	NS	2.32 ± 0.01 Aa	2.34 ± 0.01 Aa	NS	3.43 ± 0.01 Ba	3.42 ± 0.01 Ba	NS
	CD3	69.00 ± 1.06 ABa	62.49 ± 1.06 Ab	**	0.33 ± 0.01 Aa	0.32 ± 0.01 Aa	NS	0.26 ± 0.01 Aa	0.26 ± 0.01 Aa	NS	2.27 ± 0.01 Aa	2.27 ± 0.01 ABa	NS	3.44 ± 0.01 Ba	3.48 ± 0.01 Cb	*
	Anova	*	*		NS	NS		NS	NS		NS	*		**	*	
EL	CD	67.26 ± 1.45 Aa	65.00 ± 1.45 Aa	NS	0.36 ± 0.01 Aa	0.35 ± 0.01 Aa	NS	0.25 ± 0.01 Aa	0.27 ± 0.01 Aa	NS	2.24 ± 0.01 Aa	2.26 ± 0.01 Aa	NS	3.47 ± 0.02 Aa	3.54 ± 0.02 Aa	NS
	BD	65.55 ± 1.45 Ba	66.93 ± 1.45 Aa	NS	0.35 ± 0.01 Aa	0.32 ± 0.01 Ba	NS	0.29 ± 0.01 Aa	0.30 ± 0.01 Aa	NS	2.06 ± 0.01 Ba	2.27 ± 0.01 Ab	***	3.17 ± 0.02 Ba	3.63 ± 0.02 Bb	***
	APD	62.03 ± 1.45 Ca	63.23 ± 1.45 Ab	**	0.34 ± 0.01 Aa	0.33 ± 0.01 Ca	NS	0.27 ± 0.01 Aa	0.33 ± 0.01 ABa	NS	2.16 ± 0.01 Ca	2.23 ± 0.01 Ab	*	3.11 ± 0.02 Ba	3.38 ± 0.02 Cb	***
	Anova	*	NS		NS	*		NS	*		*	NS		***	*	
ML	CD	63.86 ± 1.81 Aa	65.82 ± 1.81 Aa	NS	0.36 ± 0.01 Aa	0.35 ± 0.01 Aa	NS	0.32 ± 0.01 Aa	0.29 ± 0.01 Aa	NS	2.25 ± 0.01 Aa	2.26 ± 0.01 Aa	NS	3.48 ± 0.01 Aa	3.74 ± 0.01 Ab	***
	BD	67.34 ± 1.81 Ba	63.94 ± 1.81 ABa	NS	0.36 ± 0.01 Aa	0.35 ± 0.01 Aa	NS	0.27 ± 0.01 Ba	0.28 ± 0.01 Ba	NS	2.22 ± 0.01 Aa	2.20 ± 0.01 Ba	NS	3.32 ± 0.01 Ba	3.38 ± 0.01 Ba	NS
	APD	65.50 ± 1.81 ABa	67.42 ± 1.81 Ba	NS	0.38 ± 0.01 Aa	0.37 ± 0.01 Ab	NS	0.29 ± 0.01 ABa	0.26 ± 0.01 ABa	NS	2.19 ± 0.01 ABa	2.13 ± 0.01 Ca	NS	3.42 ± 0.01 Aa	3.09 ± 0.01 Cb	***
	Anova	*	*		NS	NS		*	*		*	*		**	***	
LL	CD	68.40 ± 0.74 Aa	60.91 ± 0.74 Aa	NS	0.39 ± 0.01 Aa	0.38 ± 0.01 Aa	NS	0.27 ± 0.01 Aa	0.25 ± 0.01 Aa	NS	2.24 ± 0.03 Aa	2.14 ± 0.03 Ab	*	3.32 ± 0.01 Aa	3.12 ± 0.01 Ab	***
	BD	60.12 ± 0.74 Ba	65.90 ± 0.74 Ab	**	0.37 ± 0.01 Aa	0.35 ± 0.01 Aa	NS	0.26 ± 0.01 Aa	0.25 ± 0.01 Aa	NS	2.24 ± 0.03 Aa	2.16 ± 0.03 Aa	NS	3.30 ± 0.01 Aa	3.07 ± 0.01 Bb	***
	APD	67.24 ± 0.74 Aa	69.69 ± 0.74 Ba	NS	0.36 ± 0.01 Aa	0.36 ± 0.01 Aa	NS	0.28 ± 0.01 Aa	0.26 ± 0.01 Aa	NS	2.11 ± 0.03 Ba	2.05 ± 0.03 ABa	NS	3.06 ± 0.01 Ba	2.96 ± 0.01 Cb	**
	Anova	***	*		NS	NS		NS	NS		*	*		***	*	

PE: Pre-experimental; EL: Early lactation; ML: Middle lactation; LL: Late lactation; CD1: Control diet; BD: Broccoli by-products diet; APD: Artichoke plant by-products diet. Least square means within a column having different letters differ significantly. * *p* < 0.05; ** *p* < 0.01; *** *p* < 0.001. NS: not significant. The lowercase letters refer to the differences between days (T2 and T30) of the same type of diet in each of the rows (production); Capital letters refer to the differences between diets at each of the times for each of the rows (production).

**Table 7:** Syneresis and main components (g/100 mL) of the expelled whey from fermented milk refrigerated at 4 °C during 2 and 30 days using a mesophilic starter culture MA400.

Production/Diet		Syneresis			Fat			Protein			Lactose			Total Solids		
		T2	T30	Anova	T2	T30	Anova	T2	T30	Anova	T2	T30	Anova	T2	T30	Anova
PE	CD1	63.39 ± 0.82 Aa	67.68 ± 0.82 Ab	*	0.77 ± 0.01 Aa	0.41 ± 0.01 Ab	***	0.26 ± 0.01 Aa	0.33 ± 0.01 Ab	*	2.35 ± 0.01 Aa	2,35 ± 0.01 Aa	NS	3.23 ± 0.02 Aa	3.46 ± 0.02 Ab	***
	CD2	58.60 ± 0.82 Ba	67.97 ± 0.82 Ab	***	0.29 ± 0.01 Ba	0.45 ± 0.01 Ab	***	0.27 ± 0.01 Aa	0.33 ± 0.01 Aa	NS	2.28 ± 0.01 Ba	2.36 ± 0.01 Ab	*	3.33 ± 0.02 Ba	3.43 ± 0.02 Bb	***
	CD3	68.80 ± 0.82 Ca	67.40 ± 0.82 Aa	NS	0.23 ± 0.01 Ca	0.20 ± 0.01 Ba	NS	0.29 ± 0,01 Aa	0.33 ± 0.01 Aa	NS	2.24 ± 0.01 Ba	2.30 ± 0.01 ABa	NS	3.56 ± 0.02 Ca	3.46 ± 0.02 Ca	NS
	Anova	*	NS		*	***		NS	NS		*	*		***	**	
EL	CD	66.62 ± 0.95 Aa	64.85 ± 0.95 Aa	NS	0.24 ± 0.01 Aa	0.47 ± 0.01 Ab	***	0.26 ± 0.01 Aa	0.25 ± 0.01 Aa	NS	2.16 ± 0.02 Aa	2.21 ± 0.02 Aa	NS	3.23 ± 0.02 Aa	3.46 ± 0.02 Ab	***
	BD	59.56 ± 0.95 Ba	62.81 ± 0.95 Aa	NS	0.32 ± 0.01 Ba	0.38 ± 0.01 Bb	*	0.30 ± 0.01 Aa	0.33 ± 0.01 Ba	NS	2.17 ± 0.02 Aa	2.23 ± 0.02 Aa	NS	3.33 ± 0.02 Ba	3.43 ± 0.02 Ab	*
	APD	66.91 ± 0.95 Aa.	66.89 ± 0.95 ABa	NS	0.58 ± 0.01 Ca	0.48 ± 0.01 Ab	**	0.24 ± 0.01 ABa	0.26 ± 0.01 Aa	NS	2.19 ± 0.02 Aa	2.21 ± 0.02 Aa	NS	3.56 ± 0.02 Ca	3.46 ± 0.02 Ab	*
	Anova	**	*		**	**		*	*		NS	NS		*	NS	
ML	CD	64.26 ± 2.37 Aa	56.89 ± 2.37 Aa	NS	0.36 ± 0.01 Aa	0.33 ± 0.01 Aa	NS	0.34 ± 0.01 Aa	0.29 ± 0.01 Aa	NS	2.15 ± 0.04 Aa	2.16 ± 0.04 Aa	NS	3.27 ± 0.01 Aa	3.22 ± 0.01 Aa	NS
	BD	58.72 ± 2.37 Aa	52.49 ± 2.37 Aa	NS	0.71 ± 0.01 Ba	0.82 ± 0.01 Bb	***	0.35 ± 0.01 Aa	0.37 ± 0.01 Ba	NS	2.27 ± 0.04 Aa	2.37 ± 0.04 Ba	NS	3.74 ± 0.01 Ba	3.87 ± 0.01 Bb	**
	APD	66.38 ± 2.37 Aa	67.16 ± 2.37 Ba	NS	0.49 ± 0.01 Ca	0.37 ± 0.01 Ab	***	0.24 ± 0.01 Ba	0.19 ± 0.01 Ca	NS	2.15 ± 0.04 Aa	2.10 ± 0.04 Aa	NS	3.39 ± 0.01 Ca	3.26 ± 0.01 Ab	**
	Anova	NS	*		***	***		**	**		NS	*		**	***	
LL	CD	58.83 ± 1.20 Aa	63.39 ± 1.20 Ab	*	0.21 ± 0.01 Aa	0.27 ± 0.01 Ab	*	0.26 ± 0.02 Aa	0.24 ± 0.02 Aa	NS	2.04 ± 0.02 Aa	2.04 ± 0.02 Aa	NS	3.02 ± 0.07 Aa	3.04 ± 0.07 Aa	NS
	BD	61.42 ± 1.20 Aa	60.64 ± 1.20 Aa	NS	0.36 ± 1.20 Ba	0.25 ± 0.01 Ab	**	0.27 ± 0.02 Aa	0.24 ± 0.02 Aa	NS	2.09 ± 0.02 Aa	2.08 ± 0.02 Aa	NS	3.19 ± 0.07 Aa	3.07 ± 0.07 Aa	NS
	APD	59.48 ± 1.20 Aa	61.85 ± 1.20 Aa	NS	0.34 ± 1.20 Ba	0.45 ± 0.01 Bb	**	0.25 ± 0.02 Aa	0.23 ± 0.02 Aa	NS	1.92 ± 0.02 Ba	1.96 ± 0.02 ABa	NS	3.03 ± 0.07 Aa	3.25 ± 0.07 Aa	NS
	Anova	NS	NS		**	***		NS	NS		*	*		NS	NS	

PE: Pre-experimental; EL: Early lactation; ML: Middle lactation; LL: Late lactation; CD1: Control diet; BD: Broccoli by-products diet; APD: Artichoke plant by-products diet. Least square means within a column or row having different letters differ significantly. * *p* < 0.05; ** *p* < 0.01; *** *p* < 0.001. NS: not significant. The lowercase letters refer to the differences between days (T2 and T30) of the same type of diet in each of the rows (production); Capital letters refer to the differences between diets at each of the times for each of the rows (production).

**Table 8:** Values of total phenols and antioxidant capacity in raw milk (RM) and fermented milk manufactured by late lactation milk and stored for 30 days with milk from Murciana-Granadinas goats fed with differentiated diets, using a thermophilic (YO-MIX^TM^ 300) and mesophilic (MA400) starter cultures.

	YO-MIX^TM^ 300								MA400							
	Anova		CD		BD		APD		Anova		CD		BD		APD	
	RM		RM	LL	RM	LL	RM	LL	RM	LL	RM	LL	RM	LL	RM	LL
TPC	NS	*	49.41 a	67.56 c	50.85 a	87.45 a	45.35 a	76.82 b	NS	**	49.41 a	84.52 b	50.45 a	98.75 a	45.68 a	81.20 ba
DPPH	NS	NS	0.26 a	0.36 a	0.24 a	0.38 a	0.33 a	0.44 a	NS	*	0.51 a	0.53 a	0.38 b	0.45 b	0.47 a	0.53 a
ABTS	*	NS	0.27 b	0.22 a	0.23 b	0.20 a	0.33 a	0.24 ba	*	NS	0.26 b	0.26 a	0.24 b	0.24 a	0.33 a	0.25 a

RM: raw milk; LL: Late lactation; CD: Control diet; BD: Broccoli by-products diet; APD: Artichoke plant by-products diet; Least square means within a row having different letters differ significantly. * *p* < 0.05; ** *p* < 0.01. NS: not significant.

**Table 9:** Fatty Acids ratios and healthy indexes in raw milk and fermented milks manufactured by late lactation milk and stored for 30 days with milk from Murciana-Granadinas goats fed with differentiated diets, using a thermophilic (YO-MIX^TM^ 300) and mesophilic (MA400) starter cultures.

	Raw Milk				FMs by YO-MIX^TM^ 300				FMs by MA400			
	Anova	CD	BD	APD	Anova	CD	BD	APD	Anova	CD	BD	APD
PUFA/SFA	***	0.071 a	0.061 b	0.070 a	***	0.064 a	0.055 b	0.065 a	***	0.063 a	0.054 b	0.062 a
MUFA/SFA	**	0.41 a	0.38 b	0.39 b	*	0.41 a	0.38 b	0.40 a	**	0.40 a	0.37 b	0.39 ab
n6/n3	*	20.01 a	15.44 b	8.97 c	*	21.51 a	17.82 b	9.16 c	*	23.43 a	18.03 b	9.85 c
LA/ALA	*	18.42 a	14.19 b	8.07 c	*	20.36 a	16.59 b	8.45 c	*	22.13 a	17.02 b	9.07 c
Oleic acid/Stearic acid	***	1.57 b	1.79 b	2.25 a	***	1.58 b	1.76 b	2.27 a	***	1.67 b	1.90 b	2.37 a
∑CLA/Vaccenic acid	**	0.52 c	0.68 b	0.82 a	**	0.49 b	0.56 ab	0.69 a	***	0.48 b	0.68 ab	0.74 a
IA	***	1.90 b	2.20 a	2.18 a	**	2.01 b	2.27 a	2.21 a	***	2.05 b	2.31 a	2.26 a
IT	***	2.89 b	3.17 a	2.88 b	*	3.07 ab	3.32 a	2.96 b	***	3.04 b	3.28 a	2.97 b
HFA	***	36.64 b	40.35 a	40.51 a	***	38.99 b	42.15 a	41.97 a	***	38.39 b	41.45 a	41.61 a
HH	***	0.71 a	0.62 b	0.64 b	***	0.69 a	0.61 b	0.63 ab	**	0.69 a	0.68 a	0.63 b
HPI	***	0.52 a	0.45 b	0.45 b	***	0.49 a	0.43 b	0.45 b	***	0.48 a	0.43 b	0.44 b
DI14	**	0.019 b	0.021 ab	0.025 a	**	0.016 b	0.018 b	0.022 a	*	0.017 b	0.018 b	0.022 a
DI16	NS	0.049 a	0.045 a	0.053 a	NS	0.052 a	0.049 a	0.058 a	NS	0.047 a	0.045 a	0.055 a
DI18	***	2.01 b	2.18 ab	2.70 a	***	1.95 b	2.10 ab	2.63 a	**	2.00 b	2.18 b	2.72 a

CD: Control diet; BD: Broccoli by-products diet; APD: Artichoke plant by-products diet; Least square means within a row having different letters differ significantly. * *p* < 0.05; ** *p* < 0.01; *** *p* < 0.001. NS: not significant. PUFA/SFA (Polyunsaturated Fatty Acids/Saturated fatty acids, Equation (1)), MUFA/SFA (Monounsaturated Fatty Acid/Saturated fatty acids, Equation (2)), n6/n3 (omega 6/omega 3, Equation (3)), LA/ALA (Linoleic Acid/α-Linolenic acid, Equation (4)), Oleic acid/Stearic acid (Equation (5)), ∑CLA/Vaccenic acid (Equation (6)), IA (Aterogenicity index, Equation (7)), IT (thrombogenicity index, Equation (8)), HFA (hypercholesterolemic index, Equation (9)), HH (Hypocholesterolemic/Hypercholesterolemic ratio, Equation (10)), HPI (Health promopting index, Equation (11)), DI14 (Desaturation index 14, Equation (12)), DI16 (Desaturation index 16, Equation (13)), and DI18 (Desaturation index 18, Equation (14)).

**Table 10:** Prevalence of the most abundant volatiles (proportion of each individual peak area over total peak area, %) in fermented goat milk by different cultures.

				FMs by MA400 ^1^					FMs by YO-MIX ^TM^ 300				
	Volatile Organic Compound	Rt	Chemical Family	Anova	PE	LL CD	LL BD	LL APD	Anova	PE	LL CD	LL BD	LL APD
V1	Acetoin/2-Butanone. 3-hydroxy-	3.568	Ketone	**	1.7 a	0.0 b	0.1 b	0.0 b	**	1.7 a	0.0 b	0.0 b	0.0 b
V2	Butanoic acid/Butyric acid	4.049	Acid	**	0.5 c	1.7 b	3.6 a	4.6 a	NS	1.3	1.0	1.5	0.7
V3	Hexanal/Caproaldehyde	4.629	Aldehyde	**	6.3 a	1.3 c	5.7 a	4.8 b	***	20.7 a	4.3 c	10.2 b	10.2 b
V4	2-Heptanone/Methyl pentyl ketone	7.203	Ketone	**	0.2 b	1.0 b	3.9 a	0.8 b	**	0.2 c	4.6 a	2.6 b	1.2 bc
V5	Heptanal	7.697	Aldehyde	**	7.7 a	6.2 a	3.7 c	5.5 b	***	17.0 a	10.5 b	6.6 c	9.0 bc
V6	Dimethyl sulfone/DMSO2	8.203	Sulfur	NS	0.9	0.3	0.7	0.6	*	1.4 a	0.9 ab	0.0 b	0.0 b
V7	Benzaldehyde	10.333	Aldehyde	NS	0.4	0.4	0.3	0.2	NS	0.4	0.4	0.0	0.0
V8	Hexanoic acid/Caproic acid	11.179	Acid	***	3.8 b	2.8 b	19.4 a	18.0 a	**	2.2 c	8.4 b	9.0 b	11.2 a
V9	Octanal/Caprylaldehyde/Caprylic aldehyde	12.537	Aldehyde	**	2.9 a	1.0 b	3.4 a	3.1 a	**	9.6 a	3.3 c	6.3 b	4.0 c
V10	7-Oxabicyclo [2.2.1]heptane. 1-methyl-4-(1-methylethyl)-/p-Menthane. 1.4-epoxy	13.179	Hydrocarbon	NS	0.3	1.0	0.4	0.0	NS	nd	nd	nd	nd
V11	Benzene. 1-methyl-3-(1-methylethyl)-/b-Cymene	13.702	Terpene	NS	0.5	0.3	0.5	0.6	NS	nd	nd	nd	nd
V12	1-Hex nol-2-ethyl/2-Ethyl -1-hex nol	13.996	Alcohol	NS	6.2	6.1	6.2	4.4	**	4.5 b	9.2 a	10.6 a	7.4 ab
V13	Benzyl alcohol/a-Hydroxytoluene/Benzoyl alcohol	14.152	Alcohol	**	11.0 a	11.4 a	4.2 b	3.8 b	NS	2.6	4.8	4.9	5.1
V14	3.5-Octadien-2-one. (E.E)-	16.407	Ketone	NS	0.3	0.2	0.5	1.0	NS	0.5	0.6	0.7	1.0
V15	Octane. 1-ethoxy-/Ethyl octyl ether	16.725	Hydrocarbon	**	2.6 ab	4.2 a	1.4 b	1.7 b	NS	nd	nd	nd	nd
V16	Cyclohexene. 1-methyl-4-(1-methylethylidene)-/Terpinolene	17.403	Terpene	NS	0.2	0.4	0.0	0.0	NS	nd	nd	nd	nd
V17	2-Nonanone/Methyl heptyl ketone	17.767	Ketone	**	1.8 b	4.4 ab	6.2 a	4.2 ab	**	1.8 c	9.3 a	9.1 a	5.7 b
V18	Linalool/1.6-Octadien-3-ol. 3.7-dimethyl-/	18.34	Terpene	NS	0.2	0.2	0.0	0.0	NS	0.0	0.1	0.1	0.0
V19	Nonanal	18.662	Aldehyde	*	13.8 a	14.7 a	11.5 b	11.1 b	*	12.7 b	12.5 b	17.7 a	16.9 a
V20	Octanoic acid. methyl ester/Caprylic acid methyl ester/Methyl caprylate	19.927	Ester	NS	0.8	0.4	0.2	0.1	NS	0.2	0.1	0.1	0.2
V21	3-Cyclohexen-1-ol. 1-methyl-4-(1-methylethyl)-/1-Terpinenol	20.685	Terpene	NS	0.2	0.9	0.0	0.0	NS	0.0	0.1	0.0	0.0
V22	trans.cis-2.6-Nonadien-1-ol	22.243	Alcohol	**	7.2 ab	6.5 b	2.8 c	9.0 a	**	10.4 a	5.1 b	4.0 b	5.0 b
V23	Benzoic acid/Benzenecarboxylic acid	22.093	Acid	**	1.6 b	0.0 c	0.3 c	3.3 a	NS	nd	nd	nd	nd
V24	Octanoic acid/Caprylic acid	23.044	Acid	**	3.3 b	1.5 b	7.8 a	7.3 a	*	0.9 c	5.6 a	3.3 ab	2.0 b
V25	Butanedioic acid. diethyl ester/Succinic acid. diethyl ester	23.6	Ester	NS	0.2	0.5	0.2	0.2	NS	0.2	0.3	0.1	0.1
V26	Methyl salicylate/Benzoic acid. 2-hydroxy-. methyl ester/Salicylic acid. methyl ester	24.172	Ester	*	2.0 a	1.8 a	0.2 b	0.2 b	NS	0.4	0.4	0.3	0.5
V27	Octanoic acid. ethyl ester/Caprylic acid ethyl ester/Ethyl caprylate	24.795	Ester	NS	0.3	0.9	0.2	0.1	NS	0.3	0.1	0.0	0.3
V28	Dodecane	25.13	Hydrocarbon	**	4.5 a	2.4 b	0.6 c	0.8 c	NS	0.5	0.7	0.9	0.8
V29	Decanal/Capric aldehyde/Caprinic aldehyde	25.367	Aldehyde	NS	2.1	3.1	1.6	1.5	*	0.9 b	1.9 ab	2.5 a	2.5 a
V30	Cyclohexanol. 4-(1.1-dimethylethyl)-/4-t-Butylcyclohexanol	25.993	Alcohol	NS	0.5	0.6	0.6	0.6	NS	nd	nd	nd	nd
V31	Citronellol/6-Octen-1-ol. 3.7-dimethyl-	26.262	Terpene	*	0.2 b	1.6 a	0.2 b	0.3 b	NS	0.1	0.0	0.0	0.0
V32	Benzaldehyde. 4-(1-methylethyl)-/p-Cumic aldehyde/p-Isopropylbenzaldehyde	27.489	Aldehyde	NS	0.1	0.1	0.1	0.2	NS	nd	nd	nd	nd
V33	2-Decenal. (E)-	29.042	Aldehyde	NS	0.4	0.2	0.5	0.4	NS	1.2	0.2	0.7	0.6
V34	2-Propenal. 3-phenyl-/Cinnamaldehyde	29.454	Aldehyde	NS	2.3	4.5	3.5	2.5	NS	2.5	4.1	1.5	3.4
V35	Bornyl acetate/Bicyclo [2.2.1]heptan-2-ol. 1.7.7-trimethyl-. acetate. endo-	30.41	Terpene	NS	0.1	0.5	0.2	0.1	NS	0.1	0.2	nd	0.2
V36	2-Undecanone/Methyl nonyl ketone	31.122	Ketone	NS	0.5	0.9	0.5	0.6	NS	0.5	0.7	1.0	0.8
V47	Tridecane	31.812	Hydrocarbon	*	4.7 a	2.6 ab	1.0 b	1.2 b	NS	0.7	0.9	1.0	1.0
V38	1-Octanol. 2-butyl-/5-(Hydroxymethyl)undecane	32.881	Alcohol	NS	0.3	0.2	0.1	0.2	NS	0.1	0.2	0.1	0.4
V39	Dodecane. 4.6-dimethyl-	33.824	Hydrocarbon	NS	0.7	0.4	0.2	0.5	NS	0.4	0.2	0.1	0.2
V40	n-Decanoic acid/Capric acid/Caprinic acid	35.948	Acid	NS	0.7	0.2	0.7	0.7	*	0.0 b	1.1 a	0.0 b	0.0 b
V41	Propanoic acid. 2-methyl-. 2-ethyl-3-hydroxyhexyl ester	36.069	Ester	NS	0.6	0.0	0.0	0.0	NS	0.1	0.1	0.1	0.0
V42	.alpha.-Cubebene	36.34	Terpene	*	1.7 b	4.9 a	2.5 ab	1.9 b	*	1.5 b	2.4 a	0.8 c	2.9 a
V43	Tetradecane	38.309	Hydrocarbon	NS	1.3	1.5	0.5	0.7	NS	0.4	0.8	0.6	0.8
V44	Dodecanal/Lauraldehyde	38.696	Aldehyde	NS	0.3	0.7	0.5	0.5	NS	0.2	0.6	0.7	0.8
V45	Caryophyllene	39.011	Terpene	NS	0.5	1.2	1.0	0.5	*	0.6 ab	0.8 ab	0.3 b	1.3 a
V46	2 H-Pyran-2-one. tetrahydro-6-pentyl-/δ-Amylvalerolactone	42.491	Ketone	NS	0.5	0.6	0.5	0.4	NS	0.2	0.5	0.2	0.0
V47	2-Tridecanone/Methyl undecyl ketone	42.784	Ketone	NS	0.2	0.4	0.4	0.3	NS	0.2	0.5	0.3	0.4
V48	Pentadecane	43.026	Hydrocarbon	NS	0.7	1.1	0.3	0.3	NS	0.1	0.9	0.4	0.3
V49	Diethyl Phthalate/1.2-Benzenedicarboxylic acid. diethyl ester	45.006	Ester	NS	0.2	1.5	0.5	0.8	**	0.5 b	0.9 b	0.7 b	2.0 a
V50	Tetradecanal/Myristaldehyde	45.633	Aldehyde	NS	0.1	0.2	0.3	0.2	NS	0.1	0.3	0.3	0.2
V51	Heptadecane	47.755	Hydrocarbon	NS	0.3	0.5	0.4	0.2	NS	0.2	0.6	0.8	0.8

^1^ MA400 *Lactococcus lactis* subsp. *lactis. Lactococcus lactis* subsp. *cremoris. Lactococcus lactis* subsp. lactis biovar *diacetylactis. Streptococcus thermophilus*; YO-MIX^TM^ 300 *Streptococcus thermophilus* and *Lactobacillus delbrueckii* subsp. *bulgaricus*; nd not detected; DMS dimethyl sulphide; PE: Pre-experimental; LL: Late lactation; CD: Control diet; BD: Broccoli by-products diet; APD: Artichoke plant by-products diet. Least square means within a row or row having different letters differ significantly. * *p* < 0.05; ** *p* < 0.01; *** *p* < 0.001. NS: not significant. The lowercase letters refer to the differences between the average of PE and the fermented milk from CD, BD and APD at late lactation (LL CD. LL BD and LL APD. respectively).

## Data Availability

Data is contained within the article.
